# Single-Step Selection of Bivalent Aptamers Validated by Comparison with SELEX Using High-Throughput Sequencing

**DOI:** 10.1371/journal.pone.0100572

**Published:** 2014-06-25

**Authors:** Robert Wilson, Christian Bourne, Roy R. Chaudhuri, Richard Gregory, John Kenny, Andrew Cossins

**Affiliations:** Centre for Genomic Research at the Institute of Integrative Biology, University Of Liverpool, Liverpool, United Kingdom; Deutsches Krebsforschungszentrum, Germany

## Abstract

The identification of nucleic acid aptamers would be advanced if they could be obtained after fewer rounds of selection and amplification. In this paper the identification of bivalent aptamers for thrombin by SELEX and single-step selection are compared using next generation sequencing and motif finding informatics. Results show that similar aptamers are identified by both methods. This is significant because it shows that next generation sequencing and motif finding informatics have the potential to simplify the selection of aptamers by avoiding multiple rounds of enzymatic transcription and amplification.

## Introduction

Nucleic acid aptamers are high affinity binding molecules that have applications in diagnostics, therapy and separation science. They are normally identified by screening combinatorial (randomized) libraries of typically 10^12^–10^16^ oligonucleotides for nucleic acid sequences that bind to a chosen target molecule by a process called SELEX (Systematic Evolution of Ligands by Exponential Enrichment) that consists of multiple cycles of selection and PCR amplification.[1]–[3] In the selection step oligonucleotides compete for binding sites on the target molecule and in the amplification step the remaining pool of oligonucleotides is enriched with sequences that bind. The stabilities and/or affinities of aptamers based on natural nucleotide bases can be improved by incorporation of chemically modified bases, but these are more difficult to amplify. Aptamers based on locked nucleic acids (LNAs) would be more stable in vivo because they are resistant to nuclease enzymes, but when Vester and colleagues carried out 7 cycles of partitioning and amplification with a library that contained LNA bases they found the ratio of these bases to natural bases decreased in every cycle of amplification.[4] Gold and colleagues have used SELEX to identify high affinity aptamers based on chemically modified DNA bases, but these bases must be transcribed onto natural bases for amplification and reverse-transcribed back onto modified bases for selection.[5]–[7] These complications would be avoided if it was possible to identify aptamers without multiple cycles of selection and amplification.

Several groups have developed alternatives to SELEX based on single-step methods of selection.[8]–[11] Krylov and colleagues used Non-SELEX to identify aptamers that inhibit an enzyme that promotes cell division.[8] A combinatorial library of single stranded DNA was incubated with the target substance and unbound sequences were eliminated by capillary electrophoresis (CE). Oligonucleotides that remained bound to the target substance and oligonucleotides that dissociated during CE were retained and subjected to a further round of CE. Oligonucleotides in the fraction that was collected after three rounds of CE were cloned and Sanger sequenced. Nitsche and colleagues used MonoLEX to identify aptamers that bound to virus particles.[9] A DNA library was applied to an affinity column with heat-inactivated virus particles attached to a solid support. The column was washed to eliminate weakly bound DNA and then physically cut into sections. DNA in the sections was amplified and pyrosequenced. While these methods show that aptamers can be identified by single-step selection they were not carried out in parallel with SELEX and therefore it is not known if they produce comparable results.

In this paper we describe the parallel selection of bivalent aptamers for thrombin by SELEX and single-step selection. Thrombin is a multifunctional serine protease that plays important roles in blood clotting.[12], [13] The dominant structural feature of the 37 kDa protein is a deep negatively charged active site and adjacent hydrophobic pocket flanked at either-end by positively charged regions known as exosites I and II. Exosite I is the binding site for multiple macromolecular and low molecular weight ligands including fibrinogen, thrombomodulin, hiridin and heparin cofactor II, and exosite II is the binding site for heparin and platelet receptor GPIb-IX-V. Interactions between all three sites mediate blood clotting. DNA aptamers that bind to both exosites are known.[14] Apt-15 (5′-GGTTGGTGTGGTTGG) binds to exosite I and inhibits the conversion of soluble fibrinogen to insoluble fibrin,[15] and Apt-29 (5′-AGTCCGTGGTAGGGCAGGTTGGGGTGACT) binds to exosite II but only has a moderate effect on the conversion of fibrinogen.[16] Mayer and colleagues have shown that the inhibition of blood clotting is enhanced when APT-15 is connected to higher affinity APT-29 by a 15 base poly-dA linker,[17] and Soh and colleagues have shown that linkers identified by selection are superior to designed linkers.[18]

## Results

**Figure 1:**
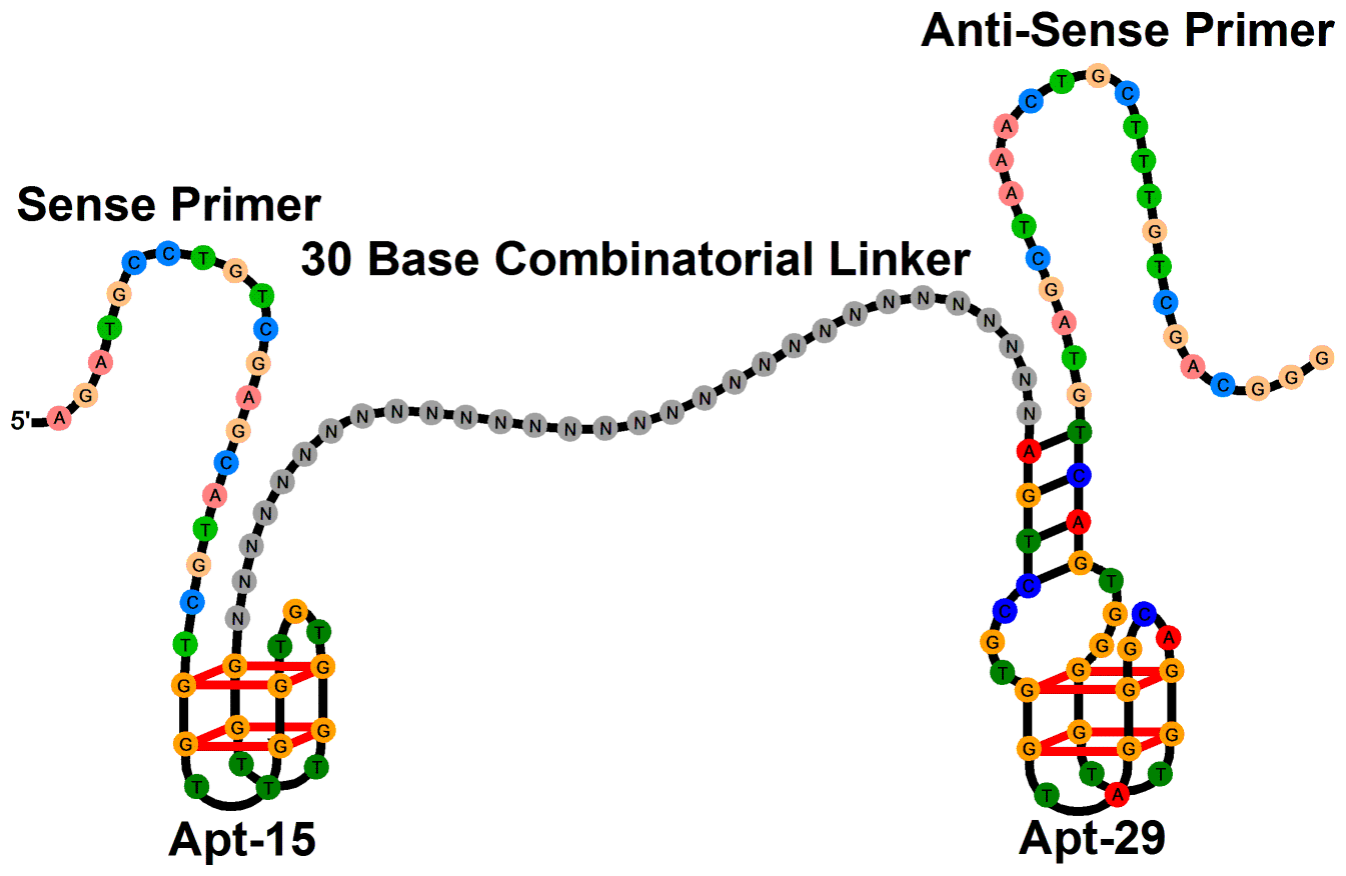
Structure of the combinatorial library that was screened for bivalent aptamers.

The structure of the DNA library screened for bivalent aptamers is shown in Figure 1. Individual oligonucleotides consist of a 30 base combinatorial (randomized) sequence bracketed by APT-15 and Apt-29, and primer sequences for PCR. The PCR primers used by us are the same as those used by Tassett and colleagues in the original selection of APT-29.[16] Selection was carried out with magnetic beads coated with thrombin conjugated to biotin polyethylene glycol (biotin-PEG). Before conjugation thrombin was characterized with antibodies and aptamers, and by MS, electrophoresis and western blotting as described previously.[14] The conjugation method was designed to minimize the number of biotin-PEG molecules per molecule of thrombin. Counter-selection was carried out with a mixture consisting of equal amounts of uncoated streptavidin beads, beads coated with biotin-PEG and beads coated with human serum albumin (HSA). HSA was characterized and biotinylated in the same way as thrombin.

The workflow for SELEX is shown in Figure S1. Five nanomoles of thermally conditioned library (3×10^15^ oligonucleotides) was slow-tilt rotated with thrombin-coated beads in 50 ml of HEPES buffer containing 0.1 mg ml^−1^ HSA and 1 µM poly(deoxyinosinic-deoxycytidilic) acid (poly-IC) for one hour, and then the beads were magnetically precipitated and washed with buffer. DNA bound to the beads was amplified with a 5′-biotin antisense primer for the number of cycles required to produce an intense band at 119 bp in PAGE (Figure S2a) and then products of this length were extracted by preparative electrophoresis on 2% agarose (Figure S2b). Extracted products were converted to single stranded DNA with streptavidin magnetic beads,[19] and fed into the next round of selection. Stringency was increased in each round of selection by decreasing the DNA concentration and amount of thrombin-coated beads, and counter selection was carried out in rounds 3 and 5.

**Figure 2:**
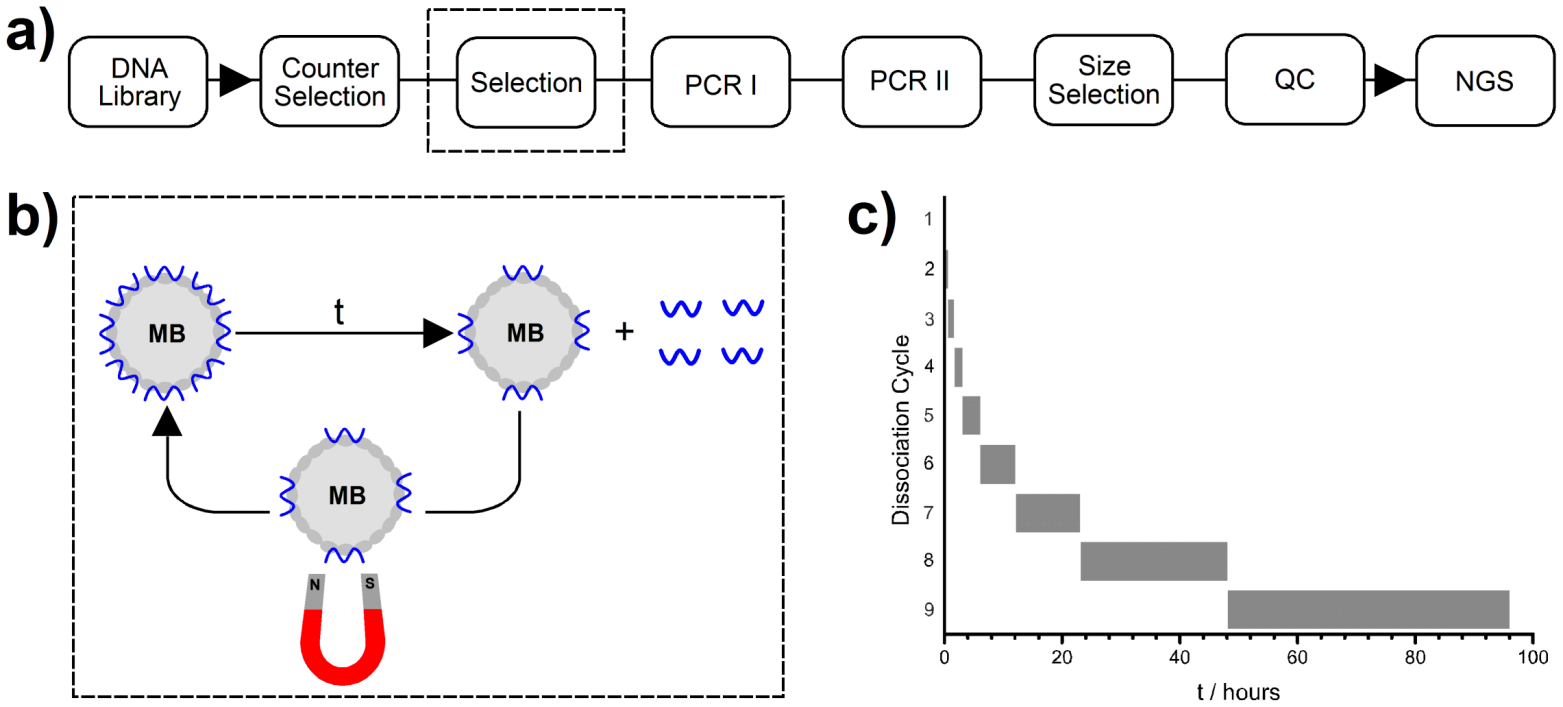
Workflow of single step selection. **a)** Key: size selection  =  preparative electrophoresis; QC  =  quality control; NGS  =  next generation sequencing. **b)** Detail of one cycle of dissociation. Thrombin-coated magnetic beads (MBs) with bound DNA are incubated for time **t** and then magnetically precipitated. DNA in the supernatant was extracted and fed into PCR I, and the MBs were re-suspended for a new cycle of dissociation. **c**) Bar-chart showing durations (**t**) of the dissociation cycles.

The workflow for single-step selection is shown in Figure 2a. Five nanomoles of library in buffer was rotated with the counter-selection mixture for 24 hours. The counter-selection mixture was precipitated and the supernatant was rotated with thrombin-coated beads for 48 hours. The beads were then precipitated and re-suspended in HEPES buffer for nine cycles of dissociation (Figure 2b). The durations of the cycles are shown in Figure 2c; the total time for all cycles (96 hours) is equivalent to 50× the half-life of a binding molecule with a dissociation rate constant (*k*_d_) of 1×10^−4^ s^−1^; monoclonal antibodies have *k*_d_ values in the range 10^−2^−10^−4^ s^−1^ and APT-29 has *k*_d_ values in the range 0.82−2.86×10^−3^ s^−1^.[20]–[22] At the end of each dissociation cycle the beads were precipitated and the supernatant was retained. DNA was extracted from the supernatants with charge-switch magnetic beads and amplified for 20 cycles of PCR. DNA still bound to the beads after 9 dissociation cycles was amplified by heating the beads to 95°C for 15 minutes followed by addition of polymerase and 15 cycles of PCR. PCR products were visualized by PAGE (Figure S3).

**Figure 3:**
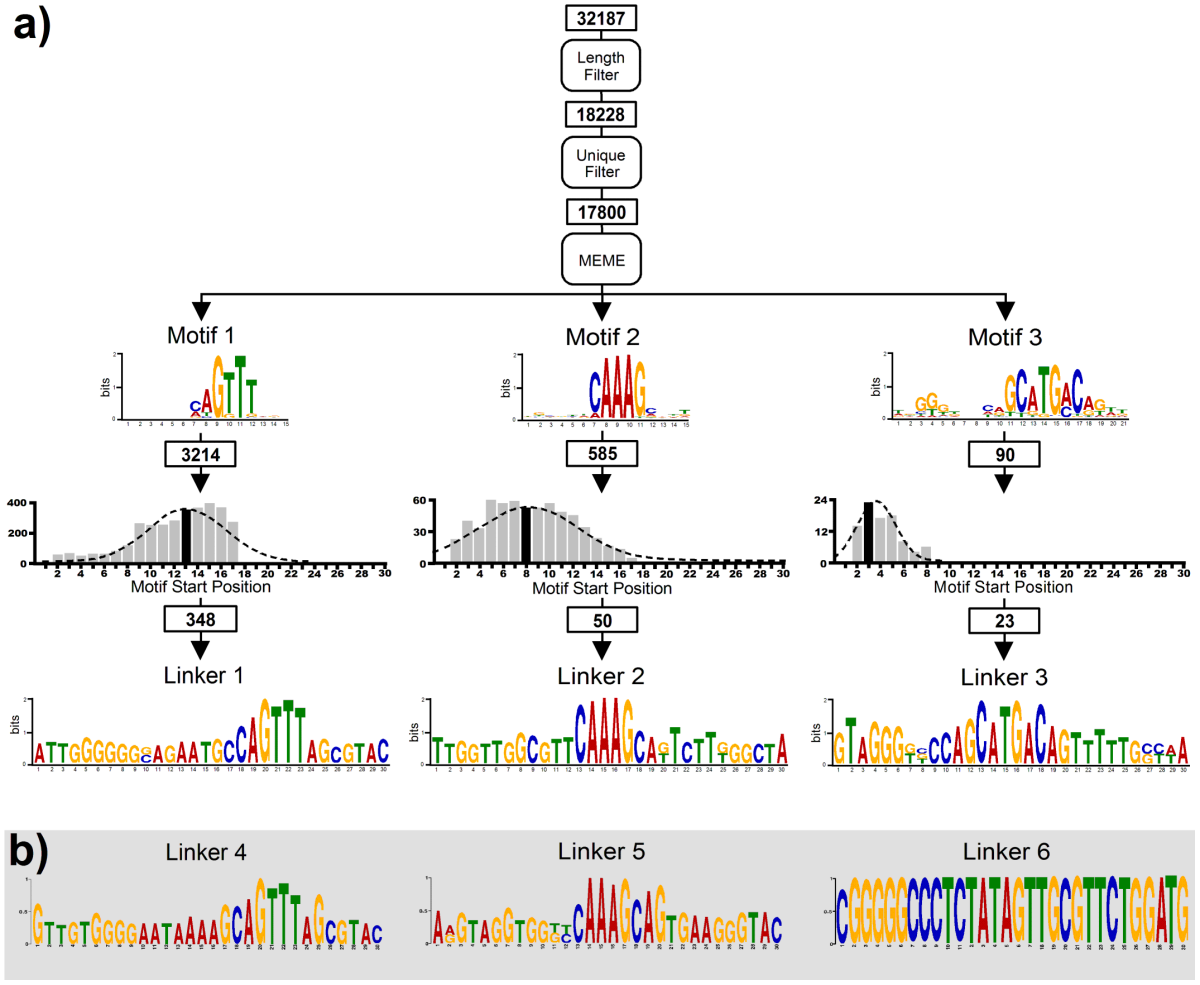
Extraction of aptamer sequences from NGS reads. **a)** Informatics pipeline as applied to DNA retained on the beads in single-step selection. Key: numbers in rectangles show the number of sequences at each stage in the pipeline. **b)** Linkers 4–6 identified in round 5 of SELEX.

**Figure 4:**
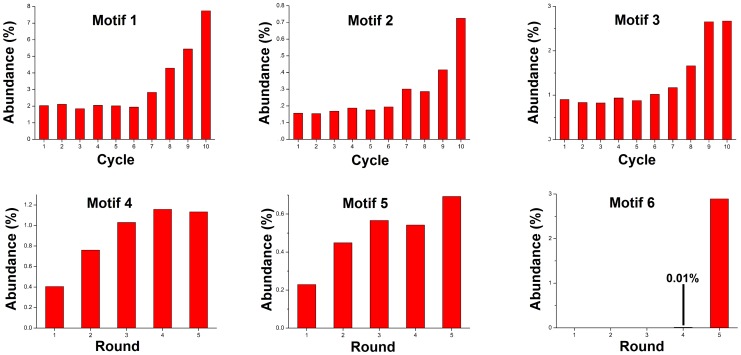
Bar graphs showing enrichment of motifs 1–3 in each cycle of single-step selection, and motifs 4–6 in each round of SELEX.

Samples from SELEX and single-step selection were amplified with barcoded sequencing primers using nested PCR for the number of cycles required to produce an intense band at 189 bp, and extracted by preparative electrophoresis on 2% agarose as shown in Figure S4. Extracted products were quantified and profiled by micro-electrophoresis as shown in S5 and S6. Next generation sequencing (NGS) was carried out on a 454 GS FLX platform using titanium chemistry according to manufacturer's instructions. Reads were sorted by barcode; an average of 28,640 nested sequences was obtained from each cycle of single-step selection, and an average of 34,299 sequences from each round of SELEX. Sequences were processed as shown in Figure 3. Any sequences not 30 bases long were discarded (length filter) and duplicate sequences were discarded in the single-step method (unique filter), but not in SELEX. The remaining sequences were searched for motifs with MEME4.9.0 downloaded onto a Linux machine and executed locally.[23] Figure 3a shows the three most abundant motifs in DNA still bound to the beads in the single-step method. The location of each motif in the 30 base sequence was plotted against its abundance (number of sequences with motif divided by total number of sequences) and then the consensus sequence with the motif located at the mean position was determined with Clustal Omega.[24] Figure 3 shows that consensus sequences based on the two most abundant motifs (linkers 1 and 2) in the single step method are very similar to consensus sequences based on the two most abundant motifs (linkers 4 and 5) in SELEX. The consensus sequence based on the third most abundant motif in the single-step method (linker 3) is a blend of linkers 1 and 2. Figure 4 shows how the abundance of motifs changed in each cycle of dissociation and each round of SELEX. In the single-step method most enrichment occurred in the last 4 cycles, but no peak was reached suggesting that further enrichment could be achieved by additional cycles. The initial increase of motifs 4 and 5 followed by a plateau is typical of SELEX,[25] and shows that enrichment was complete after three or four rounds. Linker 6 is different; it was first detected at low (0.01%) abundance in Round 4 and then increased more rapidly than any other motif in Round 5.

**Figure 5:**
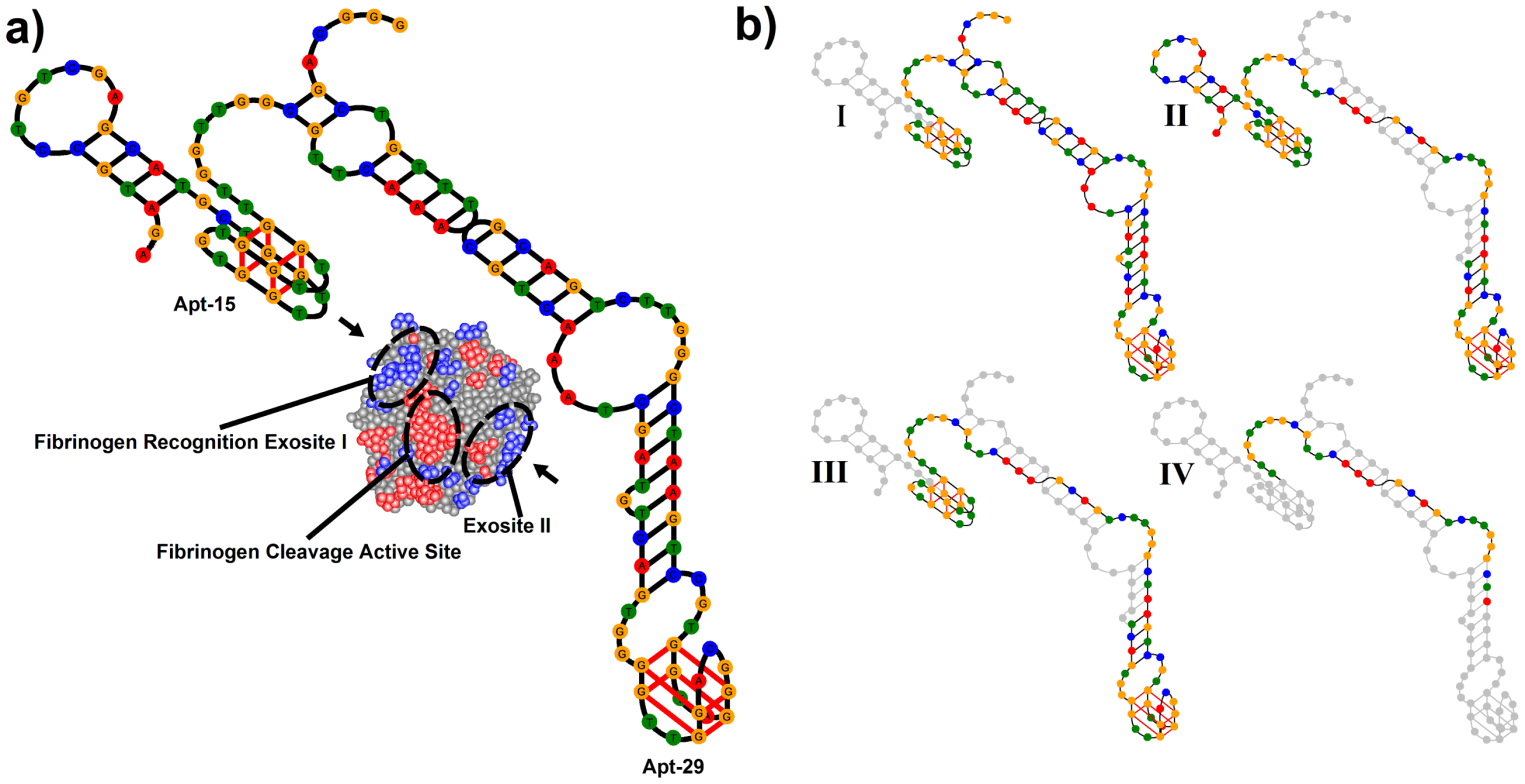
Bivalent Aptamer Structures. **a)** Predicted structure of bivalent aptamer based on Linker 2. **b)** Structures of truncated derivatives of bivalent aptamer based on linker 2; excised bases are shown as grey circles.

Lowest energy secondary structures corresponding to linker sequences bracketed by the aptamers and primer sequences were predicted with mfold (see Figures S7 and S8).[26] Table 1 shows the predicted amount of base-pairing between the aptamers and the scaffold (linker and primer sequences). The structure of the bivalent aptamer based on linker 2 is shown in Figure 5a. Structures based on linkers 1 and 3–5 are similar to structure 2, with double stranded regions formed by base-pairing between the linker and the primers. The structure of the bivalent aptamer based on linker 6 is different because there is base-pairing between the linker and APT-29. Bivalent aptamers with linkers 1–6 were investigated for their ability to inhibit thrombin-catalyzed conversion of fibrinogen to fibrin; APT-15, Apt-29 and argatroban (a small molecule inhibitor of thrombin) were also investigated. The increase in OD at 350 nm due to the thrombin-catalyzed conversion of fibrinogen to fibrin has a sigmoid profile where the duration of the initial lag-phase depends on the concentration of added the inhibitor. Inhibition curves obtained by plotting the duration of the lag-phase in the absence of the inhibitor divided by the duration in the presence of the inhibitor, against the log_10_ of the inhibitor concentration, are shown in Figures S9–S11, and affinity curves obtained by plotting absorbance at 450 nm against the log_10_ of the aptamer concentration are shown in Figures S13–S14. Table 1 shows that inhibitor concentrations required to produce half-maximal inhibition (IC_50_ values) and aptamer concentrations required to produce 50% maximal binding were similar for all bivalent aptamers. The weak affinity of APT-15 compared to its IC_50_ value is probably because the inhibition assays were homogenous, but the affinity assays involved a series of incubations and washing steps. Apt-15 is known to have a faster dissociation rate constant than Apt-29,[20]–[22] and therefore more dissociation during washing and incubation steps is expected.

**Table 1:** IC_50_ values, affinities and base pairing between scaffold and linker in bivalent aptamers.

			Base-pairing	
Aptamer	IC_50_ (nM)	50% Affinity (nM)	With Apt-15	With Apt-29
Linker 1	2.7	1.4	1	0
Linker 2	0.6	0.2	0	0
Linker 3	1.1	0.4	2	0
Linker 4	1.0	0.4	0	0
Linker 5	1.5	1.0	2	0
Linker 6	24.5	∼20	2	11
Apt-29	>1000	0.7	-	-
Apt-15	6.0	>1000	-	-
Argatroban	1.8	-	-	-
I	0.6	1.0	0	0
II	0.6	0.5	0	0
III	0.3	0.5	0	0
IV	>1000	>1000	-	-

## Discussion

An important requirement for any bivalent aptamer is the absence of internal conflict (intra-strand base-pairing) between the linker and the aptamers. Table 1 shows that all linkers selected, with the exception of linker 6, are predicted to have no base-pairing with APT-29, and low or zero base-pairing with APT-15. Only linker 6 permits base-pairing with APT-29 and this explains why the bivalent aptamer based on it has the weakest affinity and highest IC_50_ value of all the bivalent aptamers that were investigated. Three of the linkers (linkers 1, 3 and 5) permit the formation of 1 or 2 base pairs between APT-15 and the scaffold. The slightly weaker affinities and higher IC_50_ values of these bivalent aptamers compared with the bivalent aptamer based on linker 2 shows that even low amounts of internal conflict can impair function. The bivalent aptamers based on linkers 1–5 all have extensive base pairing between the linker and the primers. To understand why this was selected we obtained truncated derivatives I–IV of the bivalent aptamer with linker 2 as shown in Figure 5b, and investigated them in inhibition and affinity assays. Results shown in Figures S12 and S15, and summarized in Table 1, show that removal of base-pairing has no effect. The inhibition and affinity assays were carried out with pure aptamers, but selection was carried out in the presence of other oligonucleotides including some that would have been able to hybridize with the aptamers. All of the double-stranded regions in the bivalent aptamers stabilize APT-29 and we suggest that this protects it from disruption during selection. Thus the two main properties selected are absence of internal conflict between the linker and the aptamers, and cooperation between the linker and APT-29 to protect the latter during selection.

Our results show that single-step selection can produce similar results to SELEX. Further confirmation is provided by comparison with recent work by Soh and colleagues who started with a combinatorial library with the same general structure as in Figure 1, except that the combinatorial sequence had a length of 35 bases and the priming sequences were different.[18] After 5 rounds of high-stringency SELEX they Sanger-sequenced 10 clones. They were unable to identify any consensus motifs (we would have reached the same conclusion if we had used Sanger sequencing instead of NGS) but like us they observed base pairing between the linker and the primers. We investigated the highest affinity bivalent aptamer identified by them (TBV-08) and found that it had an IC_50_ value of 0.64 nM, which is almost identical to the bivalent aptamer based on linker 2 (Table 1). When SELEX was introduced in 1992 the most effective way to identify selected aptamers was to clone them in bacteria and sequence DNA from individual clones using first generation Sanger sequencing, but it is now possible to sequence large numbers of DNA molecules in parallel using NGS. When combined with appropriate informatics this allows aptamers to be identified after fewer rounds of selection, [27]–[29] or even, as we have shown here, after a single selection step. The latter is important because single-step selection can eliminate the multiple cycles of transcription and reverse-transcription (see reference [30] for example) that are necessary when SELEX is used to select chemically modified aptamers, and replace them with a single transcription step. Importantly, this transcription step, from a chemically-modified template to a natural-base template, is reported to be significantly more efficient than reverse transcription from a natural-base template to a chemically-modified template.[31] Therefore we anticipate that in future our approach will make it much faster and easier to select aptamers with improved properties, including those based on chemically modified nucleotides.

## Materials and Methods

### Materials

Oligonucleotides (all HPLC purified), avidin, Type 1 Gel Loading Solution, 4-hydroxyazobenzene-2-carboxylic acid (HABA), human serum albumin (HSA), fibrinogen, salmon sperm DNA, poly(deoxyinosinic-deoxycytidilic) acid (poly-IC), 10× TBE buffer and tetramethylbenzidine (TMB) solution containing 0.006% H_2_O_2_ were from Sigma. All as-supplied oligonucleotides were characterized by UV/vis spectroscopy using ODs returned by OligoCalc (http://www.basic.northwestern.edu/biotools/OligoCalc.html)[24] and PAGE. In general short oligos had concentrations that were in agreement with the supplier's data sheet, but long oligos were typically 10% less concentrated. PAGE with silver-staining of aptamers showed single bands of the correct size. 4-(2-hydroxyethyl)piperazine-1-ethanesulfonic acid (HEPES), NaCl, NaHCO_3_, NaOH, NaHPO_4_, KCl, MgCl_2_, CaCl_2_, sodium citrate, bovine serum albumin (BSA), water and Tween-20 (all molecular biology grade) were also from Sigma. Thrombin (3068 NIH units mg^−1^) was from Enzyme Research Laboratories, Swansea, UK.[14] MyOne streptavidin magnetic beads and Charge Switch magnetic beads were from Life Technologies. MyTaq HS DNA Polymerase and Reaction Buffer were from Bioline. The MinElute PCR Purification Kit was from Qiagen. EZ-Link NHS-PEG12-Biotin (biotin-PEG-NHS) and 7 k MWCO Zeba Spin Desalting Columns were from Thermo Scientific. 1.5 ml DNA Lo-Bind tubes were from Fisher Scientific. Streptavidin Peroxidase was from Abcam, Cambridge, UK. Mini-PROTEAN TGX Precast gels were from Bio-Rad. The PlusOne DNA Silver Staining Kit was from GE Healthcare.

### Equipment

UV/vis spectra were recorded on a Hewlett Packard 8452A Diode Array Spectrophotometer. Magnetic separations were carried out with either DynaMag-50 magnet or a DynaMag-2 magnet (both Life Technologies), and slow-tilt rotation was carried out with an MX2 sample mixer (Life Technologies). DNA of defined length was extracted from PCR products with a Pippin Prep preparative electrophoresis platform (Sage Science). Extracted DNA was characterized with a Qubit 2.0 Fluorometer (Life Technologies) and Bioanalyzer 2100 (Agilent). Thrombin affinity and inhibition assays were carried with a Benchmark microplate reader (Biorad).

### Biotin-PEG-Thrombin

The contents of each vial of as-supplied thrombin were dissolved in 47.5 µl of molecular grade water to give a 6.55 mg ml^−1^ solution (8.4×10^−9^ moles) in 0.1 mM sodium citrate buffer, pH 6.5, containing 0.4 M NaCl and 0.2% PEG-8000. A molar equivalent of biotin-PEG-NHS; MW  = 941.09 in 5 µl of dry DMSO was added to the solution with gentle mixing, followed immediately by 47.5 µl of 1 M sodium bicarbonate solution. The bicarbonate solution increases the pH to 8.0 and initiates aminolysis of the NHS-ester when the solution is homogenous. After gentle mixing for 1 hour, biotinylated thrombin was purified on a Zeba spin-column with water as the eluant. The concentration of thrombin in the eluate was determined using an extinction coefficient of E_280_ 1% = 18.3. HSA was biotinylated in the same way (E_280_ 1% = 5.31).

### Biotin Assay

HABA was dissolved in 10 mM NaOH to a final concentration of 2.42 mg ml^−1^. Avidin was dissolved in 50 mM sodium phosphate buffer, pH 6.0, containing 0.15 M NaCl to a final concentration of 0.5 mg ml^−1^. A 50 µM solution of biotin was prepared in 50 mM sodium phosphate buffer. A calibrator solution was prepared by adding 61.52 µl of HABA solution to 2.4 ml of avidin solution. A calibration graph was prepared by measuring the absorbance at 500 nm 10 minutes after adding 2 µl increments of biotin solution to 100 µl of calibrator solution. The concentration of biotin in biotinylated thrombin was determined by measuring the decrease in absorbance at 500 nm 10 minutes after adding 5 µl of purified biotin–thrombin to 100 µl of calibrator solution.

### Magnetic Beads For Selection And Counter Selection

MyOne streptavidin magnetic beads (Invitrogen) were washed in HEPES buffer (40 mM HEPES, 125 mM NaCl, 5 mM KCl, 1 mM, MgCl_2_, 1 mM CaCl_2_, 0.05% Tween-20, pH 7.5) and slow-tilt rotated for 1 hour with biotin-PEG-thrombin in HEPES buffer at a concentration of 40 µg of thrombin per mg of beads. Then the beads were washed with 4×1 ml of HEPES. HSA beads were prepared by rotating beads with biotin-PEG-HSA in the same way. PEG beads were prepared by rotating beads with biotin-PEG-COO^−^ (biotin-PEG-NHS that had previously been incubated in 1 M sodium bicarbonate solution overnight to hydrolyze the NHS ester.

### Single-Step: Counter Selection And Selection

A library solution was prepared by dissolving 5 nanomoles of library template (**AGATGCCTGTCGAGCATGCT** GGTTGGTGTGGTTGG ***N*** **(30)** AGTCCGTGGTAGGGCAGGTTGGGGGA **GTAGCTAAACTGCTTTGTCGACGGG**) where ***N*** **(30)** is a 30-mer combinatorial (randomized) sequence, underlined sequences are APT-15 (GGTTGGTGTGGTTGG) and APT-29 (AGTCCGTGGTAGGGCAGGTTGGGGGA), and sequences in bold font are for PCR, in 0.5 ml of HEPES buffer. The library solution was thermally conditioned at 95°C for 10 minutes, cooled to 4°C, and allowed to attain room temperature. It was then added to 49.5 ml of selection buffer (HEPES buffer containing 0.1 mg ml^−1^ HSA and 1 µM poly-IC) containing 1 mg of uncoated streptavidin beads, 1 mg PEG beads and 1 mg beads HSA beads. The beads were rotated with the library solution at room temperature for 24 hours and then precipitated for one hour on a DynaMag-50 magnet. The supernatant was transferred to a second tube and placed on a DynaMag-50 magnet for one hour. The supernatant was then transferred to a third tube to which 2 mg of thrombin beads was added and rotated for 48 hours. The beads were precipitated with a DynaMag-50 magnet and transferred to a DNA Lo-Bind tube in 1 ml of HEPES buffer and immediately precipitated with a DynaMag-2 magnet. The supernatant was discarded and the beads were washed with 3×1 ml of HEPES. The beads were them suspended in beads 1 ml of HEPES and immediately precipitated with the supernatant retained as Supernatant 1. Other supernatants were obtained in the same way at the times listed in Table 2. After removing Supernatant 9 the beads were suspended in 400 µl of water and retained.

**Table 2:** Times at which supernatants were collected in single-step selection, barcodes of sequencing primers (see Table 4) that were used to amplify DNA from these supernatants, and number of nested PCR cycles (***n***) used to attach primers (see Table 4 for sequences of sequencing primers).

Supernatant	Time	Sense Primer	Antisense Primer	*n*
1	0 minutes	MID 1	MID 8	3
2	30 minutes	MID 2	MID 10	3
3	90 minutes	MID 3	MID 11	3
4	3 hours	MID 4	MID 13	3
5	6 hours	MID 5	MID 14	6
6	12 hours	MID 1	MID 8	6
7	24 hours	MID 2	MID 10	6
8	48 hours	MID 3	MID 11	6
9	96 hours	MID 4	MID 13	9
Beads	96 hours	MID 5	MID 14	3

### Single-Step: Extraction Of DNA From Supernatants

1 ml of retained supernatant from a vial on the DynaMag-2 magnet was added to 200 µl of charge-switch purification buffer and 20 µl of charge-switch magnetic beads in a new Lo-Bind tube. After rotating for 15 minutes the beads were precipitated and the supernatants discarded. The beads were washed with 2×150 µl of charge-switch wash solution and re-suspended in 20 µl of 10 mM Tris buffer, pH 8.5.

### Single-Step: PCR Amplification Of DNA From Supernatants

20 µl aliquots of Tris buffer from charge-switch beads were mixed with 50 µl of PCR solution I containing primers and Reaction Buffer, and 30 µl polymerase solution containing 5 units of DNA Polymerase in molecular grade water to give final concentrations of 1× Reaction Buffer (1 mM dNTPs, 3 mM MgCl_2_) and 1 µM primers (sense primer: 5′-AGATGCCTGTCGAGCATGCT; antisense primer: biotin-5′-CCCGTCGACAAAGCAGTTTAGCTAC). The mixture was then amplified (60 s at 95°C; 20 cycles of 95°C for 15 seconds, 60°C for 15 seconds, 72°C for 10 seconds; final extension at 72°C for 60 s). PCR products were characterized by mixing 4∶1 with loading buffer followed by electrophoresis of 10 µl aliquots on 15% polyacrylamide gels in TBE buffer for one hour at 200 V, and silver-staining with a PlusOne DNA Silver Staining Kit according to the supplier's instructions.

### Single-Step: PCR Amplification Of DNA Retained On Magnetic Beads

20 µl aliquots of beads in water were mixed with 50 µl of PCR solution I. The mixture was heated to 95°C for 15 minutes and then cooled to 50°C for five minutes. Then the solution was mixed with 30 µl of polymerase solution and amplified (60 s at 95°C; 15 cycles of 95°C for 15 seconds, 60°C for 15 seconds, 72°C for 10 seconds; final extension at 72°C for 60 s). Products were characterized by electrophoresis.

### SELEX: First Round Selection

5 nanomoles of thermally conditioned library template in 0.5 ml of HEPES buffer was added to 49.5 ml of selection buffer containing 200 µg of thrombin beads and rotated for one hour at room temperature. The beads were then precipitated for one hour on a DynaMag-50 magnetic separator and washed with 4×1 ml HEPES buffer on a DynaMag-2 magnetic separator. The beads were then re-suspended in 340 µl of molecular grade water.

### SELEX: PCR Amplification Of DNA Retained On Magnetic Beads In First Round Selection Step

20 µl aliquots of beads in water were mixed with 50 µl of PCR solution I. The mixture was heated to 95°C for 15 minutes and cooled to 50°C for 5 minutes. Then 30 µl of polymerase solution was added and the mixture amplified (60 s at 95°C; 20 cycles of 95°C for 15 seconds, 60°C for 15 seconds, 72°C for 10 seconds; final extension at 72°C for 60 s).

### SELEX: Strand Separation Of PCR Products

8×100 µl of pooled PCR products were transferred into 4×30 µl of 10 mM Tris-HCl buffer, pH 8.0, with a MinElute PCR Purification Kit according to the supplier's instructions. 119 bp DNA was extracted with a Pippin Prep by applying each 30 µl aliquot to one lane of a 2% agarose cassette (Sage Science). Extracted products were pooled and converted to single-stranded DNA (ssDNA) with streptavidin beads pre-conditioned in 20 mM NaOH as described previously.[19] The yield of single-stranded DNA was estimated using an OD of 1.0 for 0.786 µM solution.

### SELEX: Second And Fourth Round Selection Steps

Thermally conditioned ssDNA was mixed with ***B*** (see Table 3) µg of thrombin beads in selection buffer; the amounts of beads in mg and concentrations of DNA are listed in Table 3. The mixture was rotated for one hour and then the beads were precipitated and washed with 4×1 ml HEPES on a DynaMag-2 magnetic separator. The beads were then re-suspended in 2***B*** µl of molecular grade water.

**Table 3:** SELEX: Concentrations of DNA, magnetic beads and number of additional PCR cycles.

			Beads/µg				
Round	Volume/ml	DNA/nM	Thrombin (*B*)	HSA	PEG	Streptavidin	*N*
1	50.0	100	200	0	0	0	N/A
2	3.0	10	150	0	0	0	3
3	3.0	10	150	50	50	50	6
4	3.0	1	100	0	0	0	3
5	3.0	1	100	50	50	50	3

### SELEX: Third And Fifth Round Selection Steps

The method was identical to even numbered selection rounds except that single-stranded DNA was first rotated with a counter-selection mixture of HSA beads, PEG beads and streptavidin beads; the amounts of beads are listed in Table 3. After one hour counter-selection beads were removed by magnetic precipitation and the supernatant was mixed with thrombin beads and rotated for one hour.

### SELEX: PCR Amplification Of DNA Retained On Magnetic Beads In Second And Subsequent Selection Rounds

PCR amplification was carried out in two stages. In the first stage 20 µl aliquots of thrombin beads in water from the selection step were mixed with 50 µl of PCR solution I, heated to 95°C for 15 minutes and cooled to 50°C for 5 minutes. Then 30 µl polymerase solution was added and the mixture was amplified (60 s at 95°C; 10 cycles of 95°C for 15 seconds, 60°C for 15 seconds, 72°C for 10 seconds; final extension at 72°C for 60 s). PCR products were pooled and in the second stage 20 µl aliquots were added to 50 µl of PCR solution I and 30 µl polymerase solution and amplified (95°C for 60 s; ***N*** cycles (Table 3): 95°C for 15 s, 60°C for 15 s, 72°C for 10 s; final extension at 72°C for 60 s.

### Preparation Of Samples For Sequencing

20 µl aliquots of PCR I products were mixed with 50 µl of PCR solution II containing 454 sequencing primers (Table 4) and Reaction Buffer, and 30 µl polymerase solution containing 5 units of DNA Polymerase in molecular grade water to give final concentrations of 1× Reaction buffer and 1 µM primers. The mixture was then amplified (60 s at 95°C; ***n*** cycles (Tables 2 [single-step] and Table 5 [SELEX]) of 95°C for 15 seconds, 60°C for 15 seconds, 72°C for 10 seconds; final extension at 72°C for 60 s). PCR products were transferred to 10 mM Tris buffer using a MinElute PCR Purification Kit and 189 bp DNA was isolated with a Pippin Prep running a 2% agarose cassette. Isolated DNA was transferred back into 20 µl of 10 mM Tris buffer using the MinElute kit, and characterized for size by micro-electrophoresis on a Bioanalyzer 2100, and concentration with a Qubit Fluorometer.

**Table 4:** Primers for nested PCR; 454 sequencing primers in bold type with MID sequences (barcodes) underlined.

Barcode	Sequence
**MID 1**	5′-**CGTATCGCCTCCCTCGCGCCATCAGACGAGTGCGT**AGATGCCTGTCGAGCATGCT
**MID 2**	5′-**CGTATCGCCTCCCTCGCGCCATCAGACGCTCGACA**AGATGCCTGTCGAGCATGCT
**MID 3**	5′-**CGTATCGCCTCCCTCGCGCCATCAGAGACGCACTC**AGATGCCTGTCGAGCATGCT
**MID 4**	5′-**CGTATCGCCTCCCTCGCGCCATCAGAGCACTGTAG**AGATGCCTGTCGAGCATGCT
**MID 5**	5′-**CGTATCGCCTCCCTCGCGCCATCAGATCAGACACG**AGATGCCTGTCGAGCATGCT
**MID 8**	5′-**CTATGCGCCTTGCCAGCCCGCTCAGCTCGCGTGTC**CCCGTCGACAAAGCAGTTTAGCTAC
**MID 10**	5′-**CTATGCGCCTTGCCAGCCCGCTCAGTCTCTATGCG**CCCGTCGACAAAGCAGTTTAGCTAC
**MID 11**	5′-**CTATGCGCCTTGCCAGCCCGCTCAGTGATACGTCT**CCCGTCGACAAAGCAGTTTAGCTAC
**MID 13**	5′**CTATGCGCCTTGCCAGCCCGCTCAGCATAGTAGTG**CCCGTCGACAAAGCAGTTTAGCTAC
**MID 14**	5′-**CTATGCGCCTTGCCAGCCCGCTCAGCGAGAGATAC**CCCGTCGACAAAGCAGTTTAGCTAC

**Table 5:** SELEX: barcodes of sequencing primers and numbers of nested PCR cycles used to attach sequencing primers (see Table 4 for sequences of sequencing primers).

Round	Sense Primer	Antisense Primer	*n*
1	MID 1	MID 8	3
2	MID 2	MID 10	3
3	MID 3	MID 11	3
4	MID 4	MID 13	6
5	MID 5	MID 14	6

### Motif Finding Informatics

Sequencing reads were sorted into sets by barcode and then processed by removing the 5′ and 3′ aptamer sequences using cutadapt.[32] Any trimmed sequences that were not 30 bases long were discarded. Duplicate sequences were also discarded in the single-step method, but not in SELEX. The remaining sequences were searched with MEME using the command line: meme./file.txt -dna -maxsize 600000 -mod zoops -nmotifs 10 -minw 4 -maxw 40, where file.txt is a Plain Text file containing the sequences to be searched in numbered fasta format. MEME is a computational tool for discovering motifs in a group of related DNA or protein sequences. The on-line version imposes a ceiling of <2000 on the maximum number of 30 base sequences that can be searched and therefore it was downloaded onto a Linux machine. There is no upper limit to the number of sequences that can be searched locally with the downloaded version of MEME, but the computational cost of analyzing a complete set of sequences (all sequences from one cycle of single step selection, or one round of SELEX) with MEME is prohibitive and therefore complete sets were divided into sub-sets of ≤10,000 sequences to derive intermediate motifs. Each search of a sub-set produced slightly different but highly similar intermediate motifs. Sequences with similar motifs were extracted and merged in a single file that was searched to identify final motifs. The position of the final motifs in the 30 base sequence was plotted against abundance (number of sequences with motifs divided by the total number of sequences in the set) and then sequences with the motif located at the Gaussian mean were extracted. The consensus of the extracted sequences was found with Clustal Omega, and secondary structures were predicted with mfold by applying the conditions: 25°C, 125 mM NaCl and 1 mM MgCl.

### Thrombin Inhibition Assays

Thermally conditioned aptamers (Table 6) in HEPES buffer were mixed with thrombin in HEPES buffer to a final concentration of 1 nM thrombin and allowed to stand for one hour at 25°C. 225 µl of this solution was added to the wells of a plate in triplicate and then 25 µl of fibrinogen in HEPES (centrifuged for 10 minutes at 10,000×g immediately before addition) was added to each well to a final concentration of 1 mg ml^−1^. Conversion of fibrinogen to fibrin at 25°C was monitored by measuring the increase in optical density at 350 nm.

**Table 6:** Sequences of aptamers investigated in affinity and inhibition assays; the sequences of APT-15 and APT-29 in bivalent aptamers are underlined.

**Bivalent Linker 1**	5′- AGATGCCTGTCGAGCATGCTGGTTGGTGTGGTTGGATTGGGGGGCAGAATGCCAGTTTAGCGTACAGTCCGTGGTAGGGCAGGTTGGGGTGACTGTAGCTAAACTGCTTTGTCGACGGG
**Bivalent Linker 2**	5′-AGATGCCTGTCGAGCATGCTGGTTGGTGTGGTTGGTTGGTTGGCGTTCAAAGCAGTCTTGGGCTAAGTCCGTGGTAGGGCAGGTTGGGGTGACTGTAGCTAAACTGCTTTGTCGACGGG
**Bivalent Linker 3**	5-AGATGCCTGTCGAGCATGCTGGTTGGTGTGGTTGGGTAGGGGCCCAGCATGACAGTTTTTGCCAAAGTCCGTGGTAGGGCAGGTTGGGGTGACTGTAGCTAAACTGCTTTGTCGACGGG
**Bivalent Linker 4**	5′-AGATGCCTGTCGAGCATGCTGGTTGGTGTGGTTGGGTTGTGGGGAATAAAAGCAGTTTAGCGTACAGTCCGTGGTAGGGCAGGTTGGGGTGACTGTAGCTAAACTGCTTTGTCGACGGG
**Bivalent Linker 5**	5′-AGATGCCTGTCGAGCATGCTGGTTGGTGTGGTTGGAAGTAGGTGGTTCAAAGCAGTGAAGGGTACAGTCCGTGGTAGGGCAGGTTGGGGTGACTGTAGCTAAACTGCTTTGTCGACGGG
**Bivalent Linker 6**	5′-AGATGCCTGTCGAGCATGCTGGTTGGTGTGGTTGGCGGGGGCCCTCTATAGTTGCGTTCTGGATGAGTCCGTGGTAGGGCAGGTTGGGGTGACTGTAGCTAAACTGCTTTGTCGACGGG
**TBV-08**	5′-AGCAGCACAGAGGTCAGATGGGTTGGTGTGGTTGGTGAGACCTTGCATGCGACTTGGTGAGCACGTGAGAAGTCCGTGGTAGGGCAGGTTGGGGTGACTCCTATGCGTGCTACCGTGAA
**Apt-15**	5′-GGTTGGTGTGGTTGG
**Apt-29**	5′-AGTCCGTGGTAGGGCAGGTTGGGGTGACT
**Truncate I**	5′-GGTTGGTGTGGTTGGTTGGTTGGCGTTCAAAGCAGTCTTGGGCTAAGTCCGTGGTAGGGCAGGTTGGGGTGACTGTAGCTAAACTGCTTTGTCGACGGG
**Truncate II**	5′-AGATGCCTGTCGAGCATGCTGGTTGGTGTGGTTGGTTGGTTGGCGTTCAAAGCAGTCTTGGGCTAAGTCCGTGGTAGGGCAGGTTGGGGTGACT
**Truncate III**	5′-GGTTGGTGTGGTTGGTTGGTTGGCGTTCAAAGCAGTCTTGGGCTAAGTCCGTGGTAGGGCAGGTTGGGGTGACT
**Truncate IV**	5′-TTGGTTGGCGTTCAAAGCAGTCTTGGGCTA

### Aptamer Affinity Assays

Multiwell plates (Nunc Maxisorp) were coated overnight at 4°C with 100 µl per well of 0.5 µg ml^−1^ thrombin in PBS. Wells were washed with 3×250 µl of 40 mM HEPES, pH 7.5, containing 0.05% Tween-20 (HEPES-Tween) and blocked for one hour at 25°C by gentle shaking with 250 µl per well HEPES-Tween containing 1 mg ml^−1^ BSA and 0.1 mg ml^−1^ salmon sperm DNA. Wells were washed with 3×250 µl of HEPES-Tween and then 100 µl of thermally conditioned 5′-biotin aptamer (Table 6) in HEPES buffer was incubated in the wells for one hour with shaking. Wells were washed with 3×250 µl of HEPES-Tween and then 100 µl of streptavidin peroxidase diluted 1∶100 in HEPES-Tween was incubated in the wells for one hour with shaking. Wells were washed with 4×250 µl HEPES-Tween and then 200 µl of TMB solution was incubated in the wells with shaking for 10 minutes (20 minutes for Apt-15). The enzyme reaction was stopped by addition of 50 µl per well of 1 M H_2_SO_4_ and the absorbance was measured at 450 nm.

## Supporting Information

Figure S1 Scheme of SELEX. Key: size selection  =  preparative electrophoresis; PCR II =  nested PCR with sequencing primers; QC  =  quality control (quantification and micro-electrophoresis); NGS  =  next generation sequencing.
(TIF)

Figure S2 **a)** 15% poly-acrylamide gel developed at 100 V for two hours and stained with silver showing PCR products from SELEX; products produced by +3 cycles of PCR (red rectangle) were selected for preparative electrophoresis. Key: L = 20 bp ladder; white numerals  =  number PCR cycles where 10 is the product of the first stage PCR, and +3, +6, +9, +12 and +15 are the products of the second stage PCR. **b)** Results of preparative electrophoresis showing band centered on 119 bp that was extracted surrounded by white rectangles. Key: white numerals indicate lane numbers (lane 5 has calibrator DNA of lengths 20, 75, 150, 300 and 600 bp); screen E shows ethidium bromide fluorescence versus time; screen G shows a fluoresce image of the developed agarose gel.
(TIF)

Figure S3 15% poly-acrylamide gel developed at 200 V for one hour and stained with silver of DNA amplified from supernatants and retained on beads in single-step selection. Key: Lanes 1–9 show PCR products from supernatants 1–9; Lane 10 shows PCR products from DNA retained on beads.
(TIF)

Figure S4 **a)** Scheme of nested PCR for attachment of 454 sequencing primers. **b)** 12% poly-acrylamide gel developed at 200 V for 45 minutes and stained with silver showing PCR trials to find number of cycles required to produce a band at 198 bp with minimal non-specific products. In the example shown products produced by 6 cycles of PCR enclosed in red rectangle were selected for preparative electrophoresis. Key: L = 20 bp ladder; white numerals  =  number of nested PCR cycles. **c)** Preparative electrophoresis on lanes 1–4 with extraction of a band centered on 189 bp (enclosed in white rectangle). Key: white numerals indicate lane numbers (lane 5 has calibrator DNA of lengths 20, 75, 150, 300 and 600 bp); screen E shows ethidium bromide fluorescence versus time; screen G shows a fluoresce image of the developed agarose gel.
(TIF)

Figure S5 Quality control of samples from single-step selection before sequencing. Numbers in brackets are sample concentrations in ng µl^−1^ in a volume of 20 µl. Peaks at 35 and 10,350 bp in micro-electrophoresis profiles are internal calibrators.
(TIF)

Figure S6 Quality control of samples from SELEX before sequencing. Numbers in brackets are sample concentrations in ng µl^−1^ in a volume of 20 µl. Peaks at 35 and 10,350 bp in micro-electrophoresis profiles are internal calibrators.
(TIF)

Figure S7 Predicted lowest energy secondary structures of bivalent aptamers based on linkers 1–3. Nucleotides of Apt-15 are shown in red font and nucleotides of Apt-29 are shown in blue font.
(TIF)

Figure S8 Predicted lowest energy secondary structures of bivalent aptamers based on linkers 4–6. Nucleotides of Apt-15 are shown in red font and nucleotides of Apt-29 are shown in blue font.
(TIF)

Figure S9 Inhibition curves of Apt-15 and Apt-29.
(TIF)

Figure S10 Inhibition curves of bivalent aptamers.
(TIF)

Figure S11 Inhibition curves of argatroban and bivalent aptamer TBV-08.
(TIF)

Figure S12 Inhibition curves of truncated derivatives of the bivalent aptamer based on linker 2.
(TIF)

Figure S13 Affinity curves of Apt-29 and Apt-15.
(TIF)

Figure S14 Affinity curves of bivalent aptamers.
(TIF)

Figure S15 Affinity curves of truncated derivatives of the bivalent aptamer based on linker 2.
(TIF)
