# Supplementary figures and images for: Single-Step Selection of Bivalent Aptamers Validated by Comparison with SELEX Using High-Throughput Sequencing
Source: PLoS One. 2014 Jun 25;9(6):e100572. doi: 10.1371/journal.pone.0100572 (PMC4070925; doi:10.1371/journal.pone.0100572)

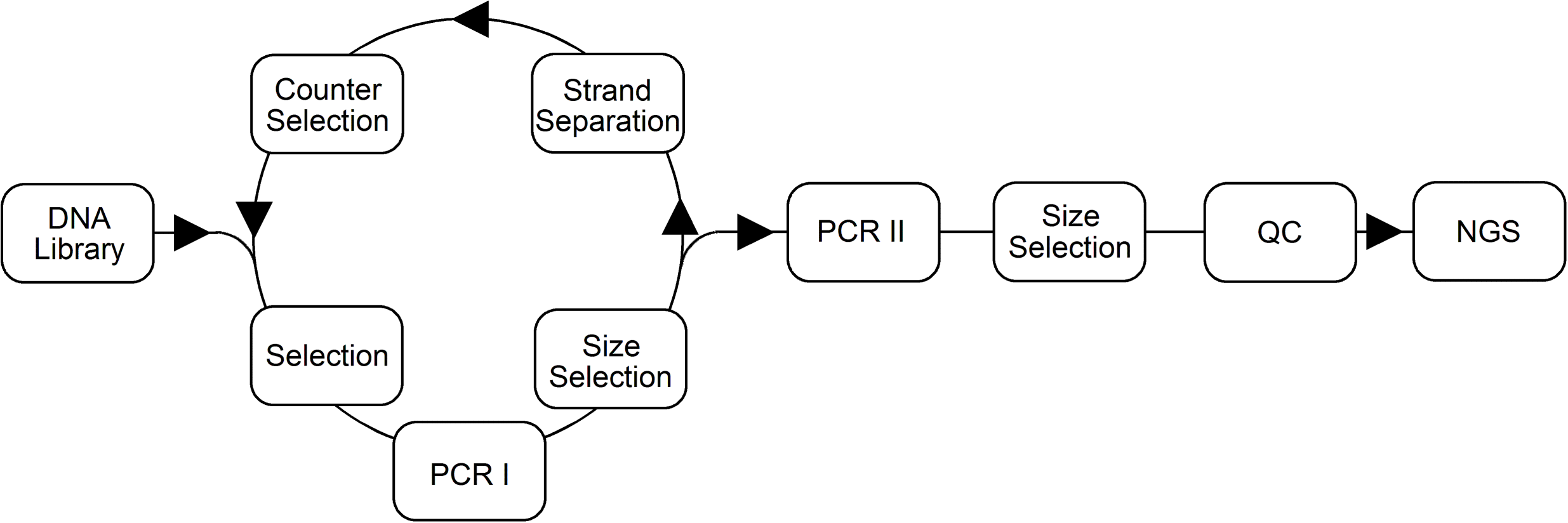

Supplement: Figure S1 — Scheme of SELEX. Key: size selection = preparative electrophoresis; PCR II = nested PCR with sequencing primers; QC = quality control (quantification and micro-electrophoresis); NGS = next generation sequencing. (TIF) [file pone.0100572.s001.tif]

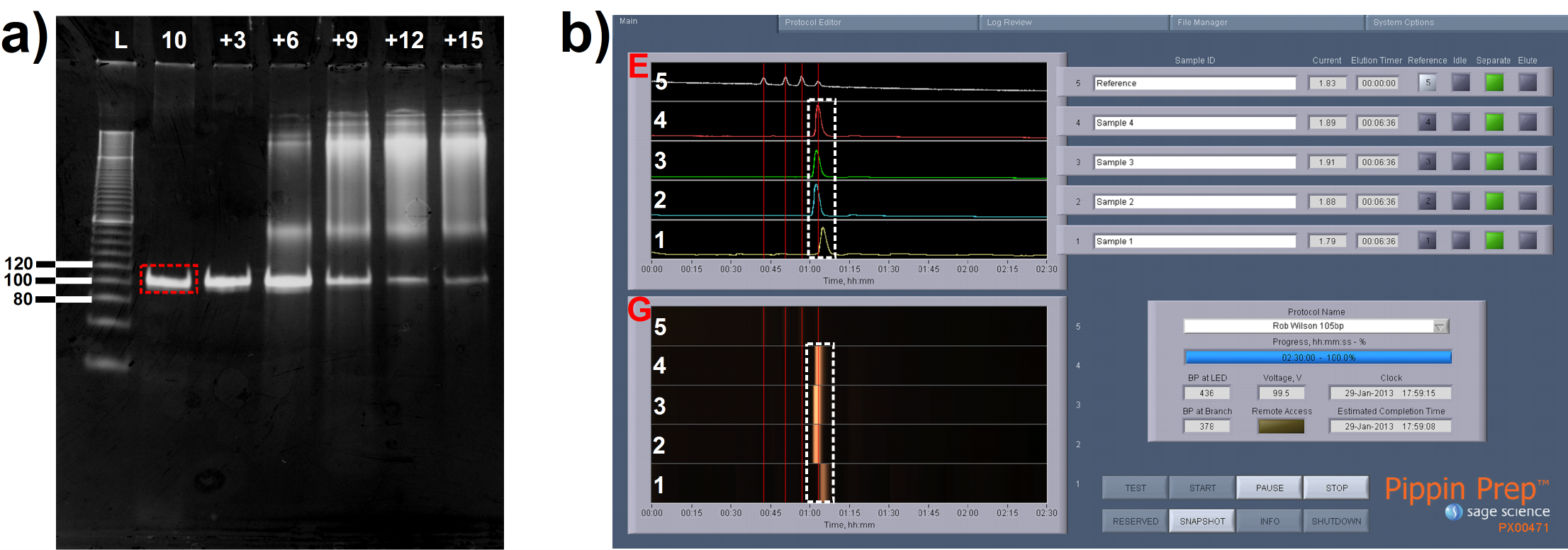

Supplement: Figure S2 — a) 15% poly-acrylamide gel developed at 100 V for two hours and stained with silver showing PCR products from SELEX; products produced by +3 cycles of PCR (red rectangle) were selected for preparative electrophoresis. Key: L = 20 bp ladder; white numerals = number PCR cycles where 10 is the product of the first stage PCR, and +3, +6, +9, +12 and +15 are the products of the second stage PCR. b) Results of preparative electrophoresis showing band centered on 119 bp that was extracted surrounded by white rectangles. Key: white numerals indicate lane numbers (lane 5 has calibrator DNA of lengths 20, 75, 150, 300 and 600 bp); screen E shows ethidium bromide fluorescence versus time; screen G shows a fluoresce image of the developed agarose gel. (TIF) [file pone.0100572.s002.tif]

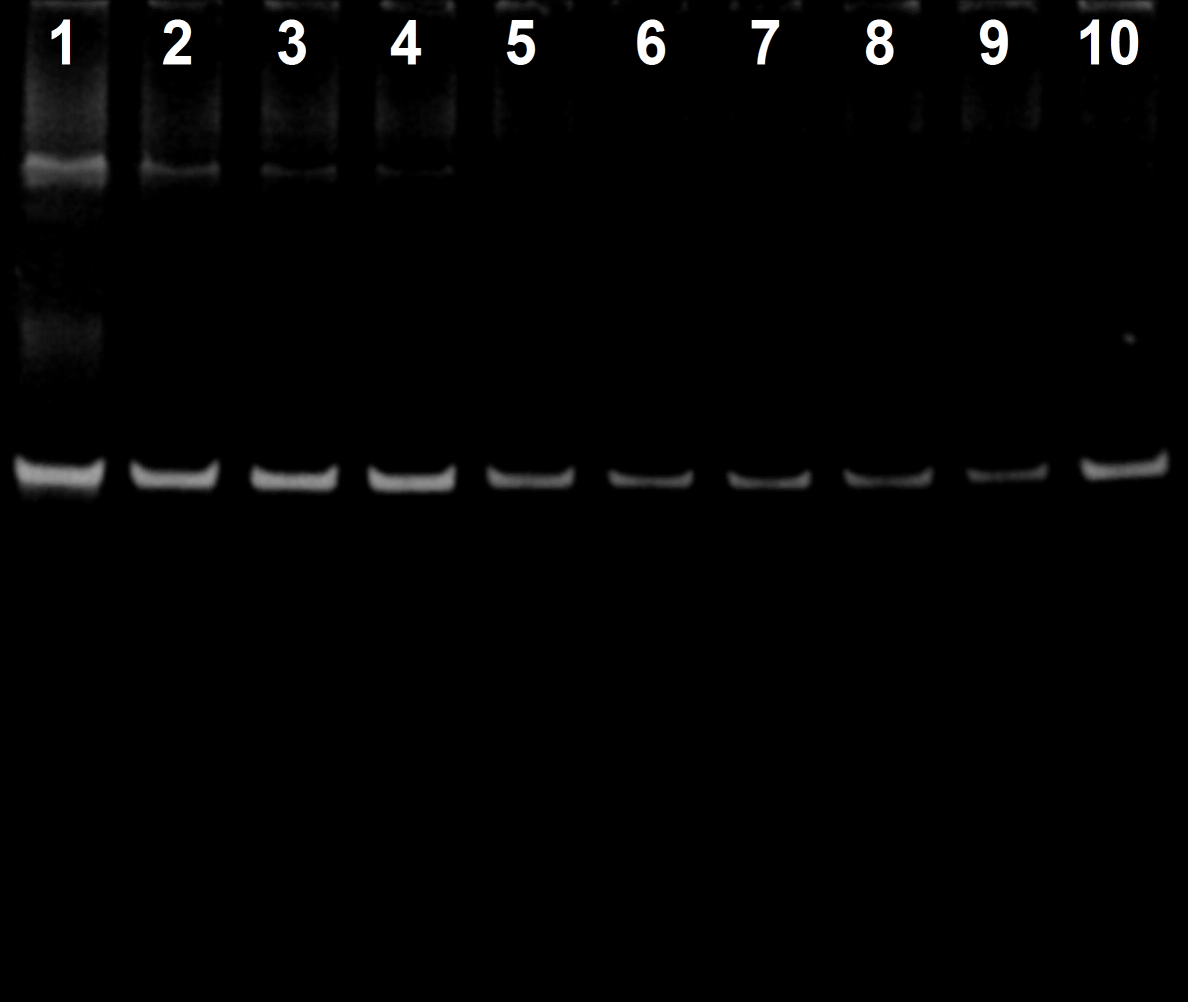

Supplement: Figure S3 — 15% poly-acrylamide gel developed at 200 V for one hour and stained with silver of DNA amplified from supernatants and retained on beads in single-step selection. Key: Lanes 1–9 show PCR products from supernatants 1–9; Lane 10 shows PCR products from DNA retained on beads. (TIF) [file pone.0100572.s003.tif]

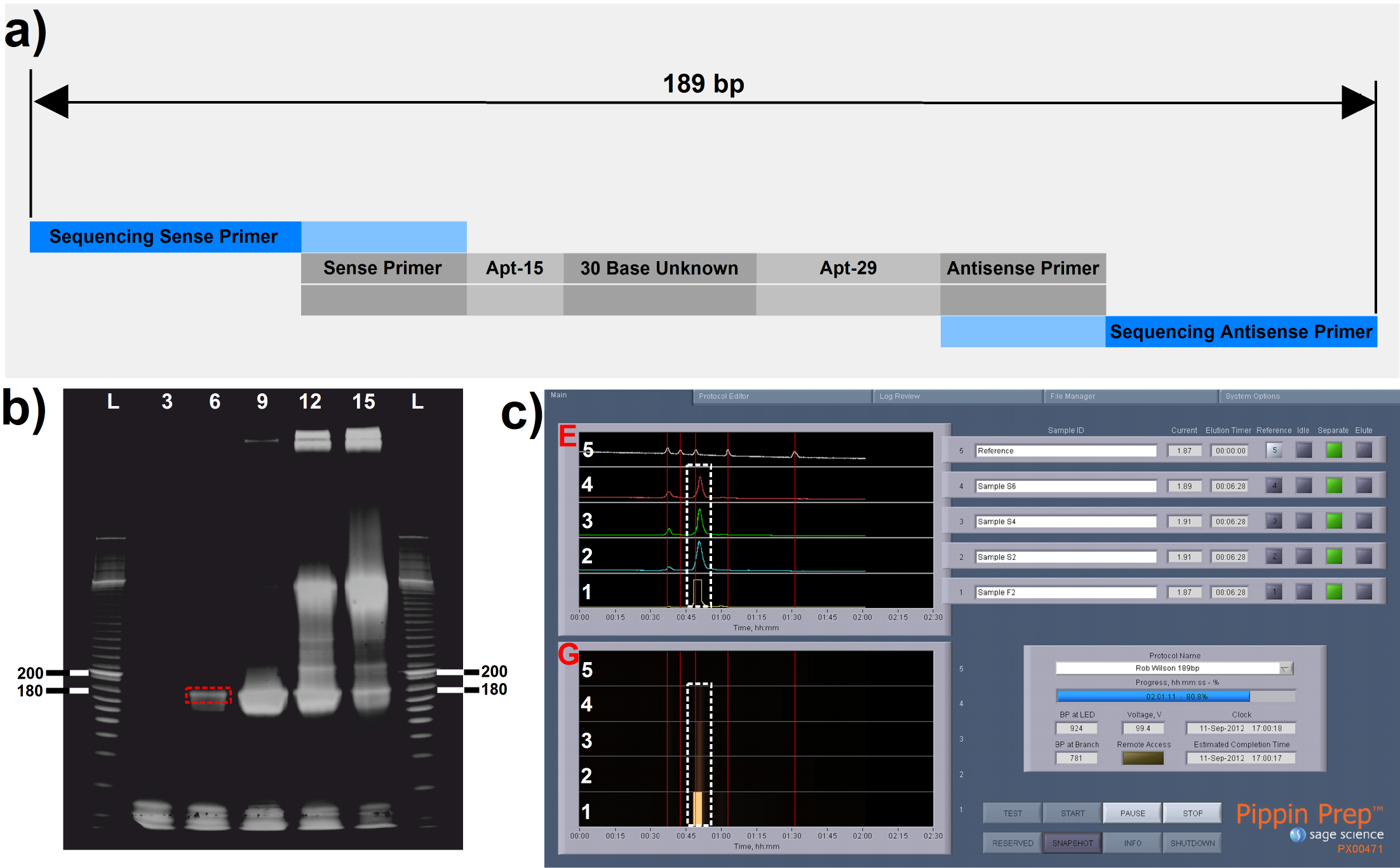

Supplement: Figure S4 — a) Scheme of nested PCR for attachment of 454 sequencing primers. b) 12% poly-acrylamide gel developed at 200 V for 45 minutes and stained with silver showing PCR trials to find number of cycles required to produce a band at 198 bp with minimal non-specific products. In the example shown products produced by 6 cycles of PCR enclosed in red rectangle were selected for preparative electrophoresis. Key: L = 20 bp ladder; white numerals = number of nested PCR cycles. c) Preparative electrophoresis on lanes 1–4 with extraction of a band centered on 189 bp (enclosed in white rectangle). Key: white numerals indicate lane numbers (lane 5 has calibrator DNA of lengths 20, 75, 150, 300 and 600 bp); screen E shows ethidium bromide fluorescence versus time; screen G shows a fluoresce image of the developed agarose gel. (TIF) [file pone.0100572.s004.tif]

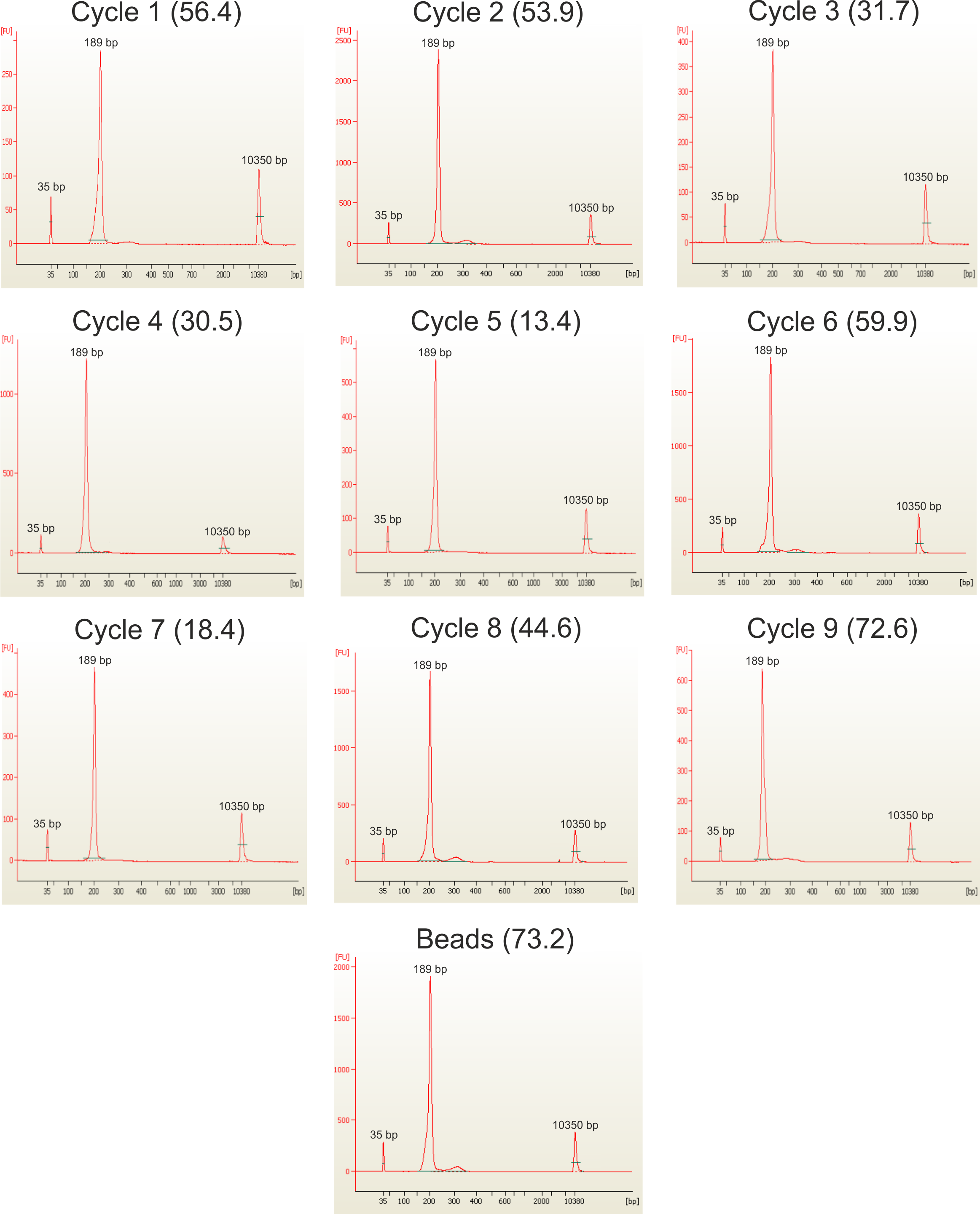

Supplement: Figure S5 — Quality control of samples from single-step selection before sequencing. Numbers in brackets are sample concentrations in ng µl−1 in a volume of 20 µl. Peaks at 35 and 10,350 bp in micro-electrophoresis profiles are internal calibrators. (TIF) [file pone.0100572.s005.tif]

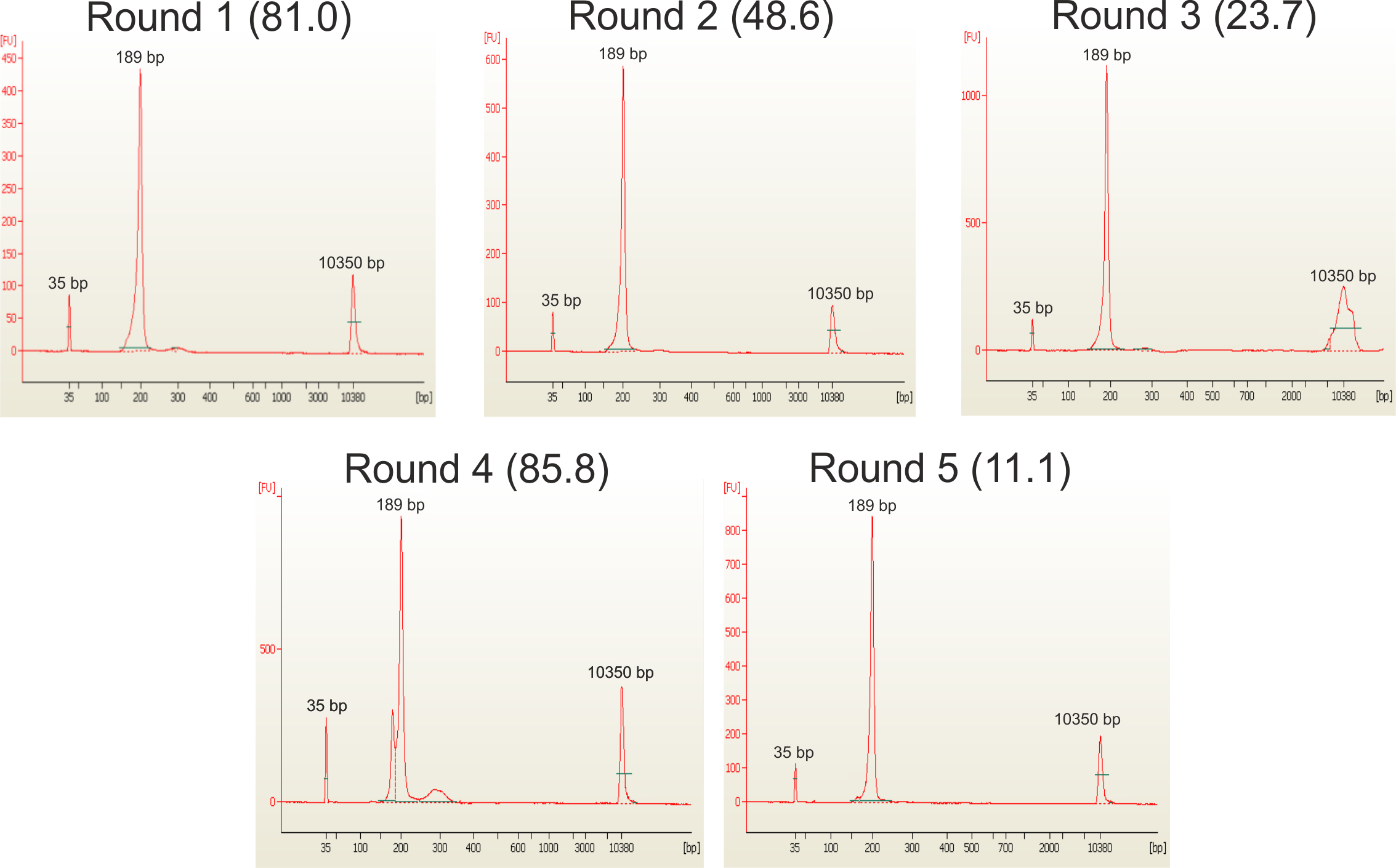

Supplement: Figure S6 — Quality control of samples from SELEX before sequencing. Numbers in brackets are sample concentrations in ng µl−1 in a volume of 20 µl. Peaks at 35 and 10,350 bp in micro-electrophoresis profiles are internal calibrators. (TIF) [file pone.0100572.s006.tif]

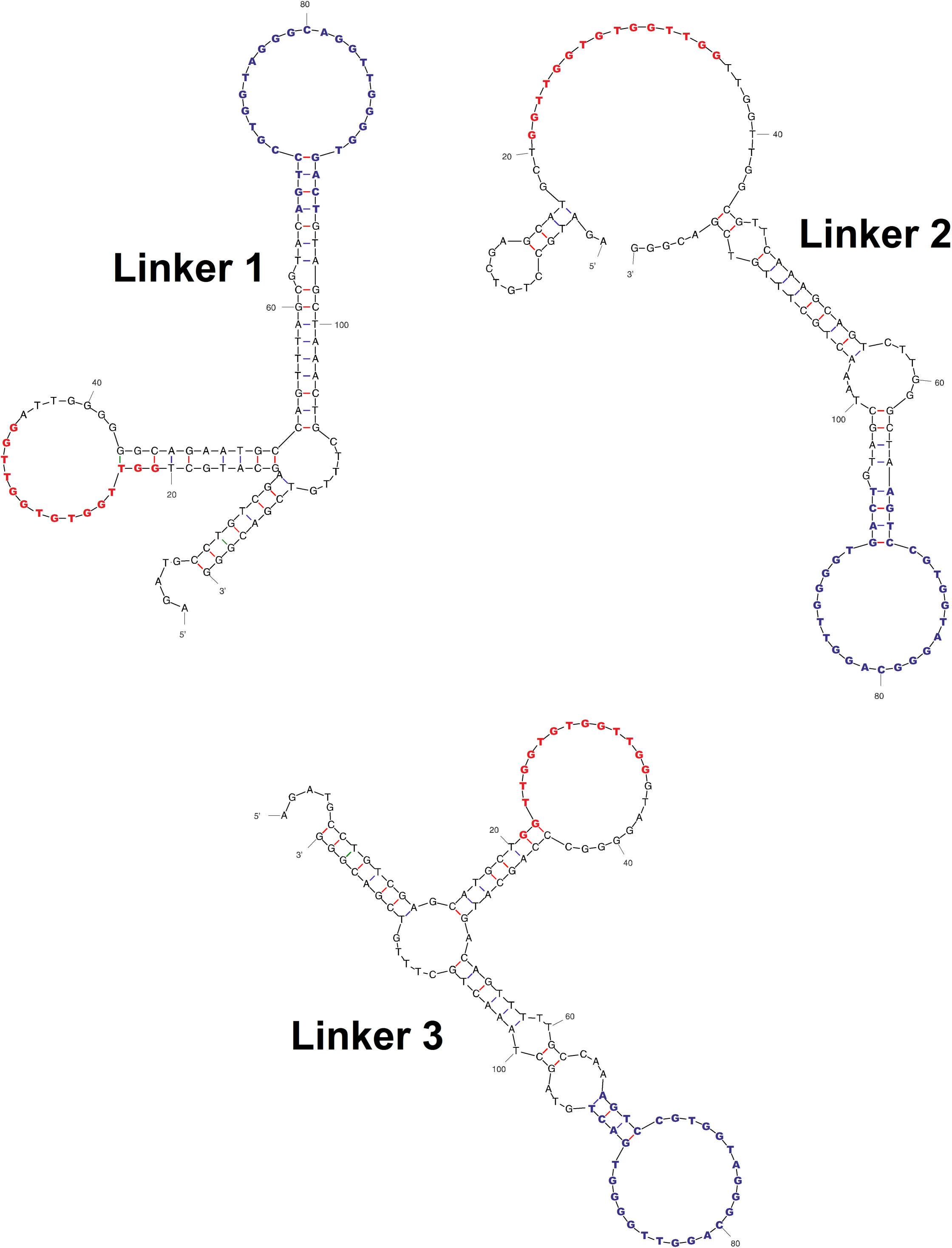

Supplement: Figure S7 — Predicted lowest energy secondary structures of bivalent aptamers based on linkers 1–3. Nucleotides of Apt-15 are shown in red font and nucleotides of Apt-29 are shown in blue font. (TIF) [file pone.0100572.s007.tif]

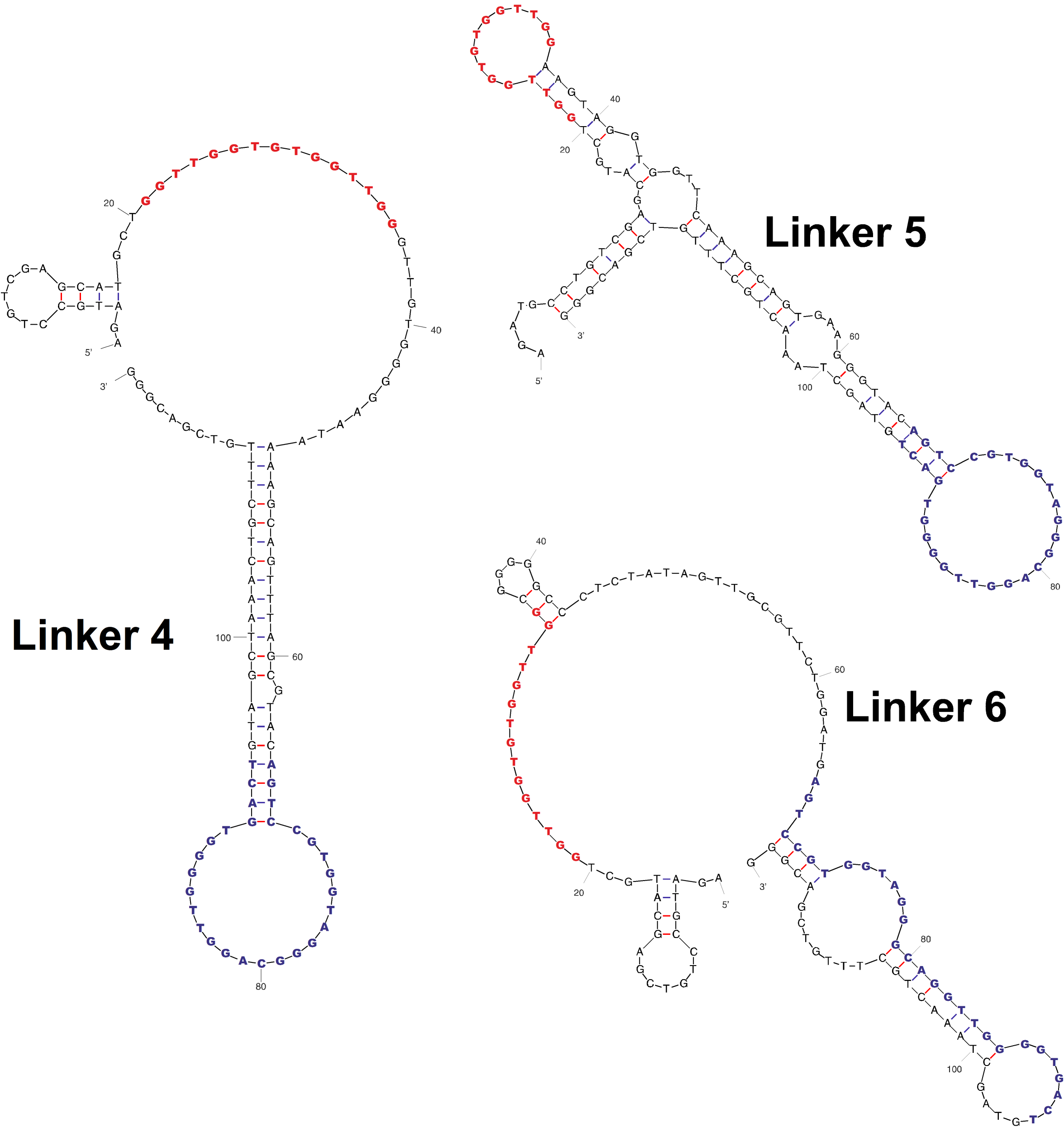

Supplement: Figure S8 — Predicted lowest energy secondary structures of bivalent aptamers based on linkers 4–6. Nucleotides of Apt-15 are shown in red font and nucleotides of Apt-29 are shown in blue font. (TIF) [file pone.0100572.s008.tif]

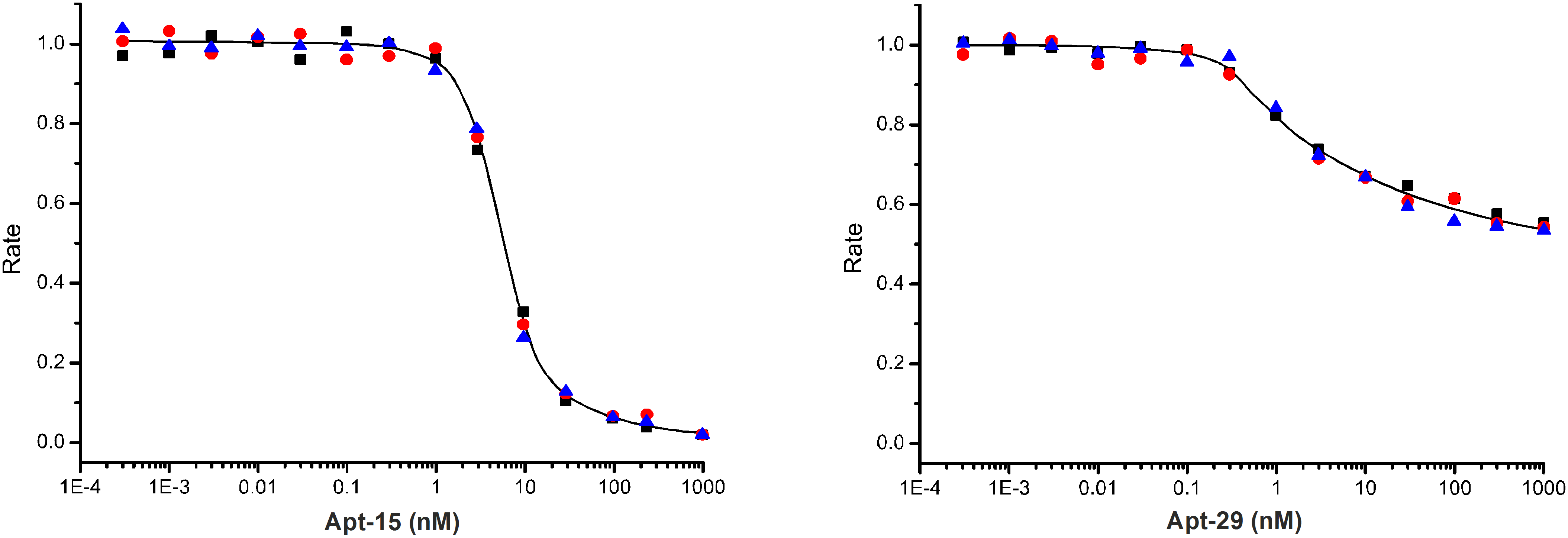

Supplement: Figure S9 — Inhibition curves of Apt-15 and Apt-29. (TIF) [file pone.0100572.s009.tif]

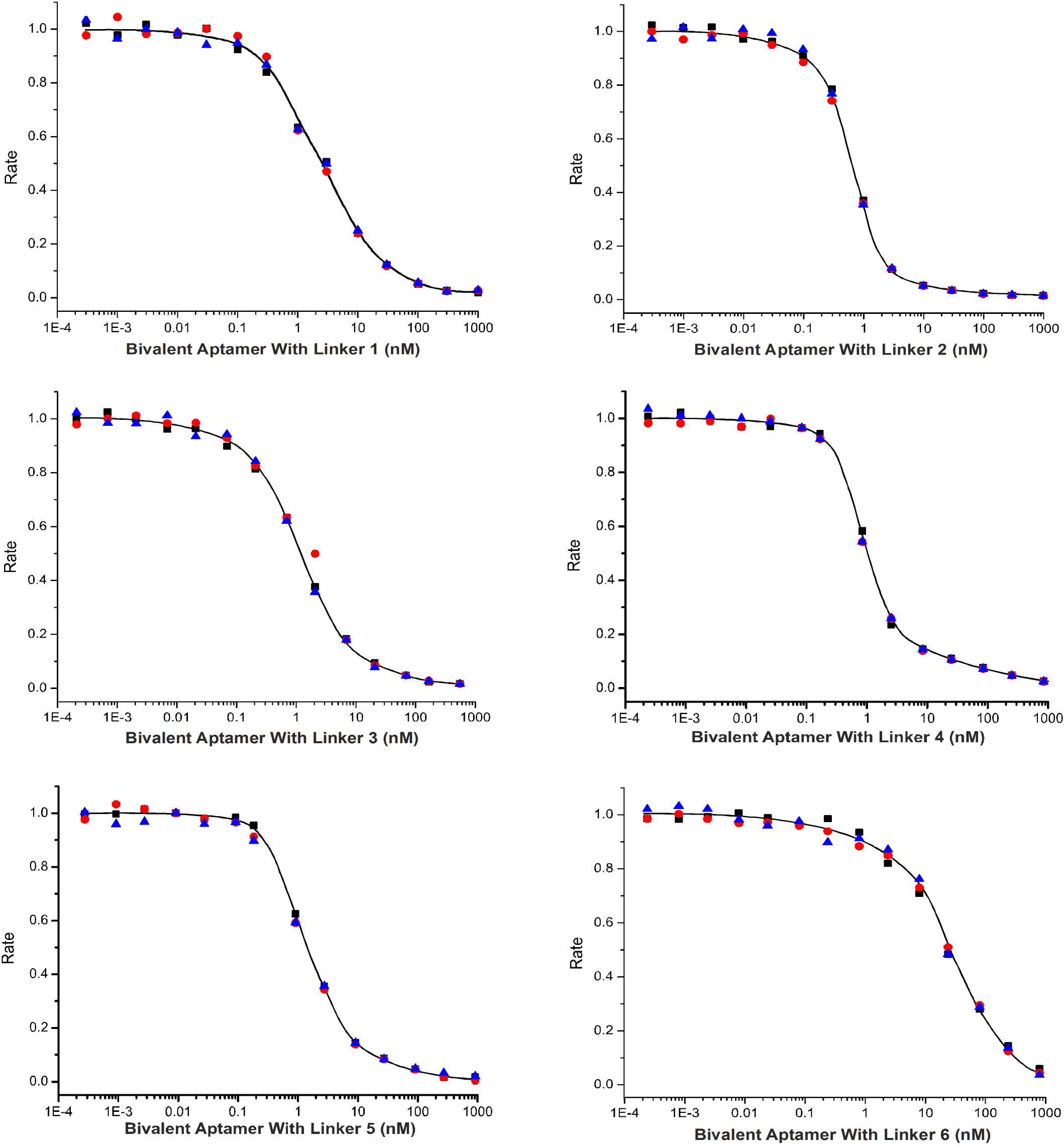

Supplement: Figure S10 — Inhibition curves of bivalent aptamers. (TIF) [file pone.0100572.s010.tif]

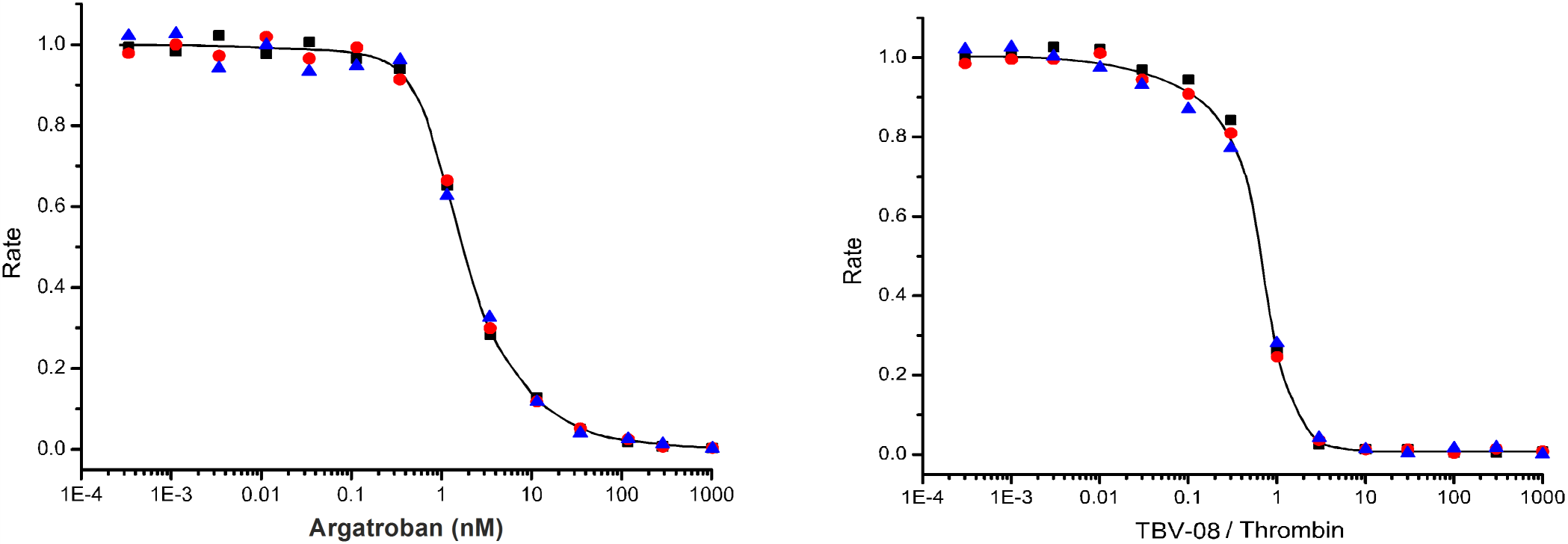

Supplement: Figure S11 — Inhibition curves of argatroban and bivalent aptamer TBV-08. (TIF) [file pone.0100572.s011.tif]

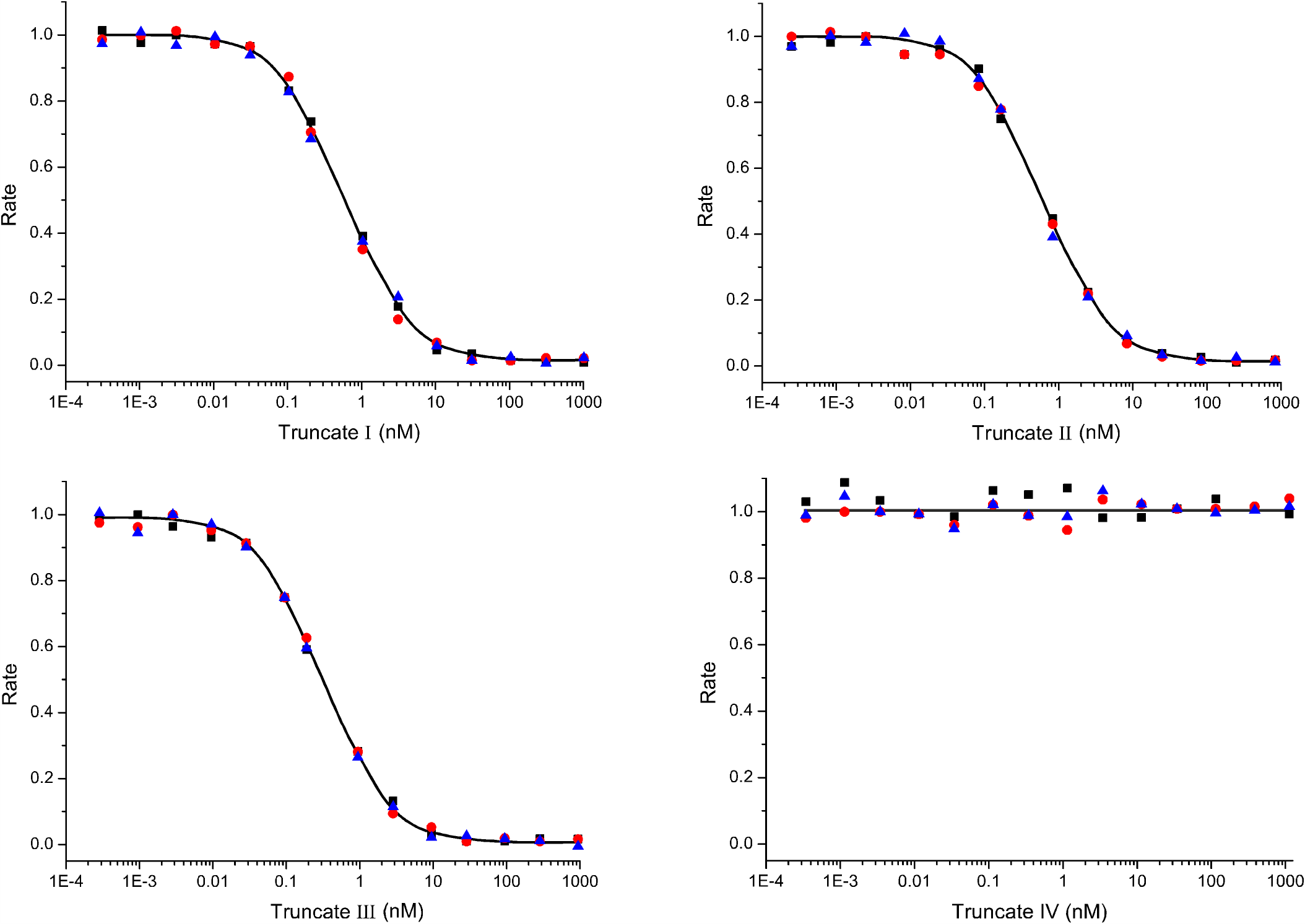

Supplement: Figure S12 — Inhibition curves of truncated derivatives of the bivalent aptamer based on linker 2. (TIF) [file pone.0100572.s012.tif]

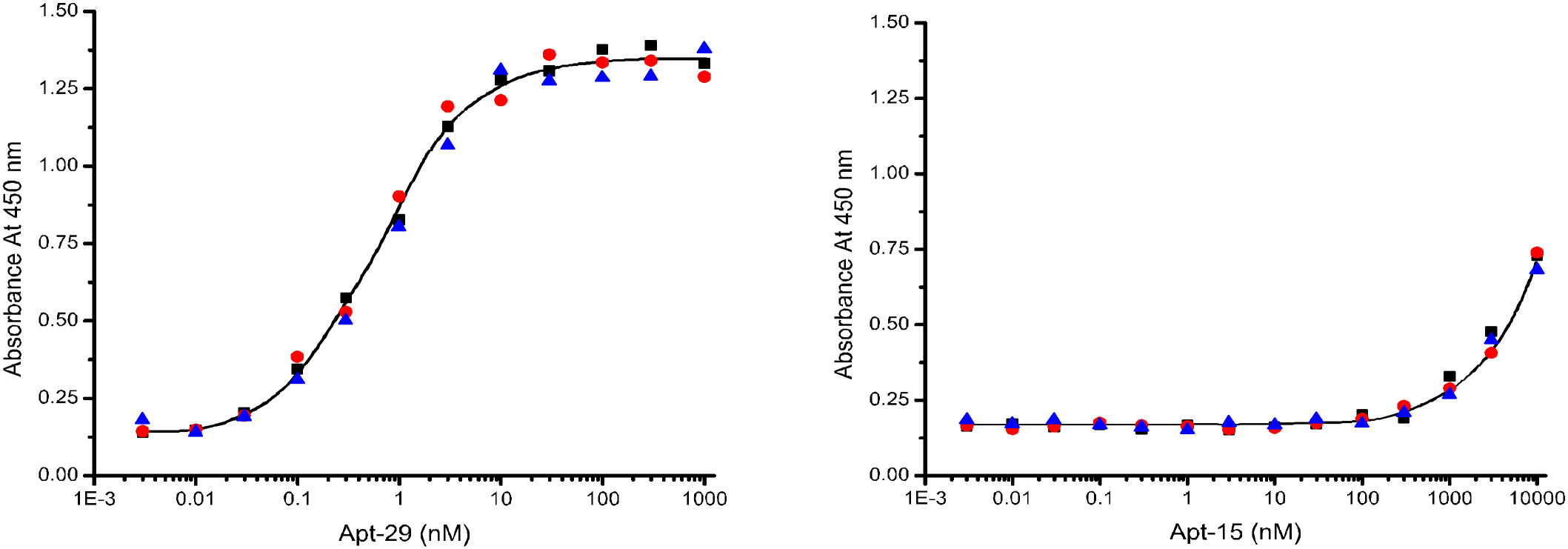

Supplement: Figure S13 — Affinity curves of Apt-29 and Apt-15. (TIF) [file pone.0100572.s013.tif]

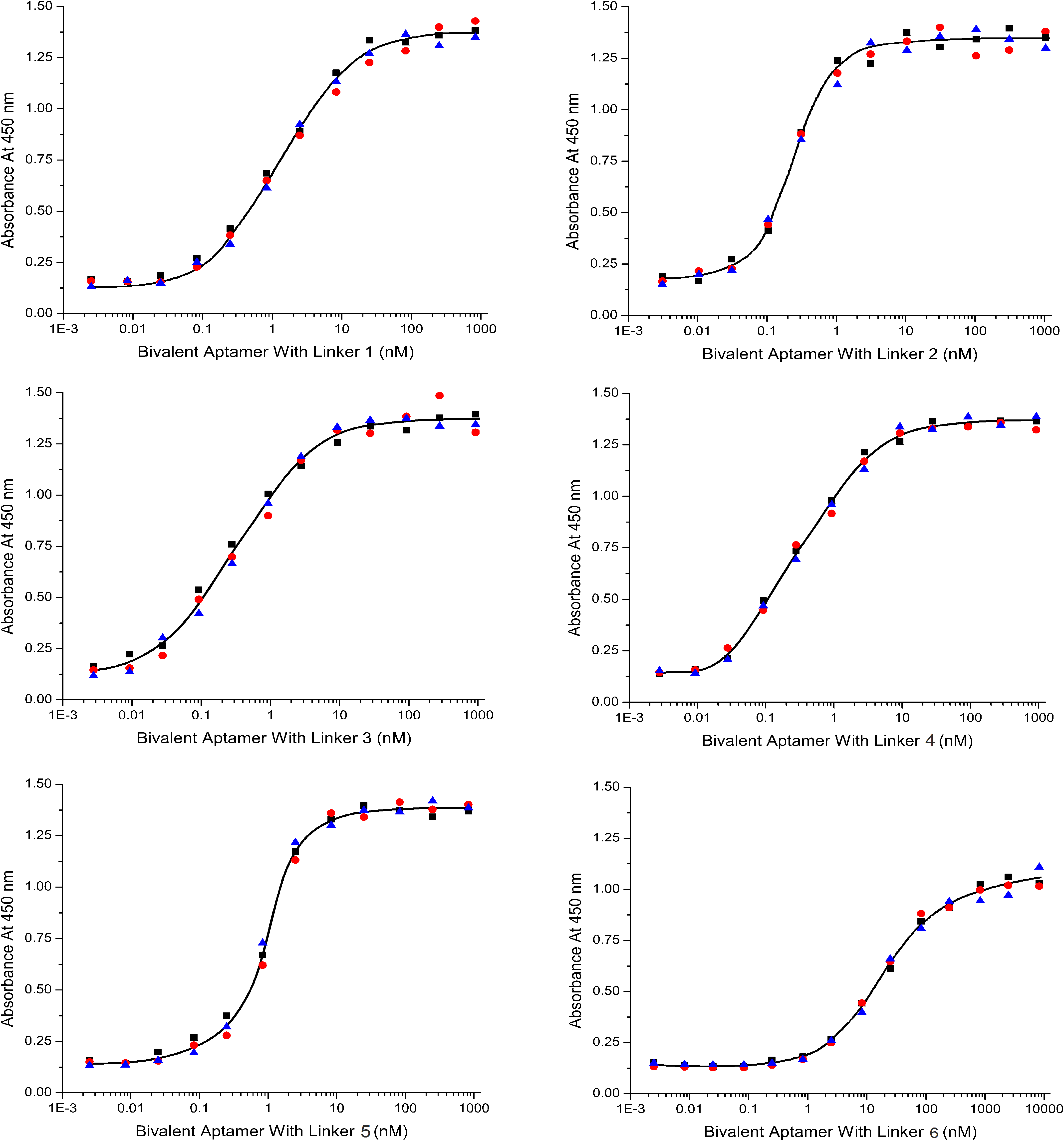

Supplement: Figure S14 — Affinity curves of bivalent aptamers. (TIF) [file pone.0100572.s014.tif]

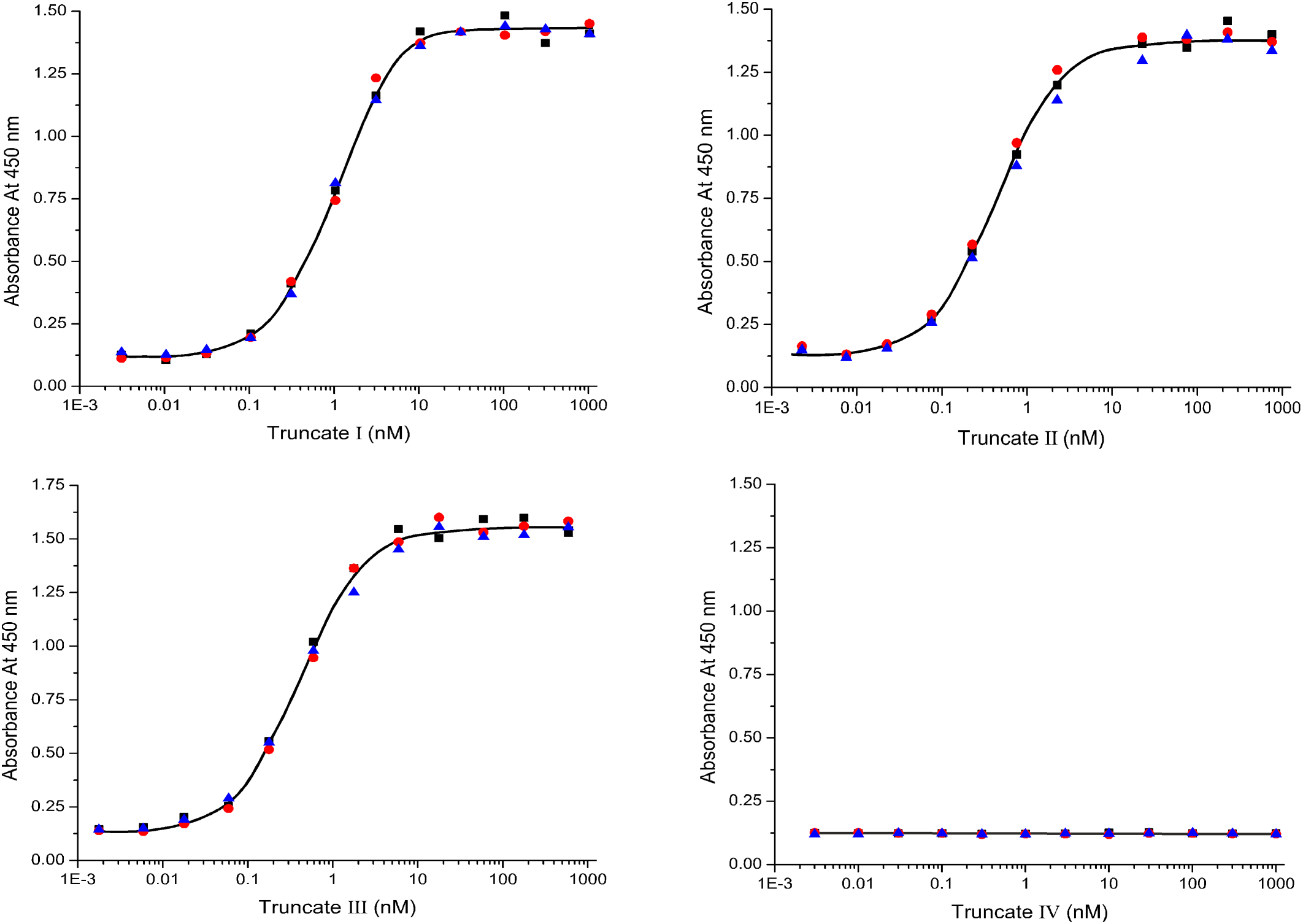

Supplement: Figure S15 — Affinity curves of truncated derivatives of the bivalent aptamer based on linker 2. (TIF) [file pone.0100572.s015.tif]
